# Environmental distribution and genomic characteristics of *Solirubrobacter*, with proposal of two novel species

**DOI:** 10.3389/fmicb.2023.1267771

**Published:** 2023-12-01

**Authors:** Zhu-Ming Jiang, Tong Mou, Ye Sun, Jing Su, Li-Yan Yu, Yu-Qin Zhang

**Affiliations:** ^1^Institute of Medicinal Biotechnology, Chinese Academy of Medical Sciences and Peking Union Medical College, Beijing, China; ^2^State Key Laboratory of Dao-di Herb, Beijing, China

**Keywords:** *Solirubrobacter*, pangenome, UV resistance, microbial resources, distribution

## Abstract

*Solirubrobacter* spp. were abundant in soil samples collected from deserts and other areas with high UV radiation. In addition, a novel *Solirubrobacter* species, with strain CPCC 204708*^T^* as the type, was isolated and identified from sandy soil sample collected from the Badain Jaran Desert of the Inner Mongolia autonomous region. Strain CPCC 204708*^T^* was Gram-stain positive, rod-shaped, non-motile, non-spore-forming, and grew optimally at 28–30°C, pH 7.0–8.0, and in the absence of NaCl. Analysis of the 16S rRNA gene sequence of strain CPCC 204708*^T^* showed its identity within the genus *Solirubrobacter*, with highest nucleotide similarities (97.4–98.2%) to other named *Solirubrobacter* species. Phylogenetic and genomic analyses indicated that the strain was most closely related to *Solirubrobacter phytolaccae* KCTC 29190*^T^*, while represented a distinct species, as confirmed from physiological properties and comparison. The name *Solirubrobacter deserti* sp. nov. was consequently proposed, with CPCC 204708*^T^* (= DSM 105495*^T^* = NBRC 112942*^T^*) as the type strain. Genomic analyses of the *Solirubrobacter* spp. also suggested that *Solirubrobacter* sp. URHD0082 represents a novel species, for which the name *Candidatus* “Solirubrobacter pratensis” sp. nov. was proposed. Genomic analysis of CPCC 204708*^T^* revealed the presence of genes related to its adaptation to the harsh environments of deserts and may also harbor genes functional in plant-microbe interactions. Pan-genomic analysis of available *Solirubrobacter* spp. confirmed the presence of many of the above genes as core components of *Solirubrobacter* genomes and suggests they may possess beneficial potential for their associate plant and may be important resources for bioactive compounds.

## 1 Introduction

The genus *Solirubrobacter*, belonging to the family *Solirubrobacteraceae*, the order *Solirubrobacterales*, the phylum *Actinomycetota*, was first identified by Singleton et al. (2003) with *Solirubrobacter pauli* as the type species. Two other species, *Solirubrobacter soli* and *Solirubrobacter ginsenosidimutans*, were subsequently reported to be identified from soil samples in ginseng fields in 2007 (Kim et al., 2007). And in 2014, two additional species (*S. phytolaccae* and *S. taibaiensis)* were identified after isolation from the roots and stems of *Phytolacca acinosa* Roxb., respectively (Wei et al., 2014; Zhang L. et al., 2014). These five currently known species generally inhabit soil and plant-based ecosystems.^1^

The boom in cultivation-independent techniques and multi-omics technologies enable improved studies on the diversity of yet-uncultivated microorganisms, also termed Microbial Dark Matter (MDM) (Jiao et al., 2020). It was found that the genus *Solirubrobacter* abundantly inhabits an Indian desert, just below members of the genera *Gaiella* and *Streptomyces* (Sivakala et al., 2018). In five different types of natural soil ecosystems in Northwest China, *Solirubrobacter* was a dominant genus (Zhang et al., 2019). It was also noted that members of the *Actinomycetales* and *Solirubrobacterales* are more abundant in soils with lower organic carbon available in varied agricultural landscapes (Shange et al., 2012).

Besides *Solirubrobacter* show potential for applications such as plant growth promotion and discovery of unique bioactive compounds and activities (Franke-Whittle et al., 2015). *S. ginsenosidimutans* DSM 22325*^T^*, for instance, displayed ginsenoside conversion activity, transforming ginsenoside Rb1 into ginsenoside F2, a phytochemical with numerous pharmacological properties (Kim et al., 2021; Zhou et al., 2021).

Desert ecosystems worldwide are abundant and diverse sources of microbiota. Harsh conditions such as extreme ultraviolet radiation, carbon and nitrogen scarcity, limited energy, hyper-aridity, and extreme temperatures, characterize these ecosystems. However, these stressful ecosystems host remarkably diverse microbial communities. Therefore, deserts can be viewed as microbial resource hotspots (Bull et al., 2016; Leung et al., 2020). One primary stress factor in deserts is high ultraviolet radiation (UVR), which all desert inhabitants, including plants, have to mitigate. Interestingly, *S. pauli* JCM 13025*^T^*, was reported to be a radiation-resistant strain, which has drawn considerable research attention (Singleton et al., 2003).

So far, the genus *Solirubrobacter* has been detected in soils (Sánchez-Marañón et al., 2017), biocrust (Miralles et al., 2020), rhizosphere habitats of various crops (Aguiar et al., 2020; Feng et al., 2021; Lee et al., 2021) and medicinal plants (Dong et al., 2018). It is also proposed that *Solirubrobacter* spp. may be pioneering organisms that enable microbiome develop in plant rhizospheres, thereby playing important roles in maintaining host plant health in ecologically stressful environments (Sivakala et al., 2018; Zhang et al., 2019). Its potential as plant-promoting, UV-resistant microorganisms is regarded as beneficial for desert ecosystem health, especially in the context of changing climate patterns.

In the current study, we investigated the ecological distribution of *Solirubrobacter* across different environmental samples, including desert soils. The research also resulted in isolation of *Solirubrobacter* strains leading to the discovery of a new species. Furthermore, we also conducted a global pan-genomic analysis of the genus *Solirubrobacte*r to evaluate the functional and biological resource potential.

## 2 Materials and methods

### 2.1 Sample collection

Twelve rhizosphere soil samples from herbs (IMB15101S–IMB15112S) were collected from high-altitude barren hills in Xinjiang. And another twelve rhizosphere soil samples from Yunnan Ethno-Medicinal plants (IMB21101S–IMB21112S) were collected from Ailao mountain in Yunnan. While desert soil samples (IMB19101D–IMB19112D, IMB15201D–IMB15212D, IMB16101D–IMB16112D) were collected from the Gurbantunggut Desert, the Tengger Desert and the Badain Jaran Desert, respectively. Cow feces samples (IMB20301F–IMB20312F) were collected from Fangshan in Beijing, while crow feces samples (IMB20101F–IMB20112F) were collected from Nanhaizi Wetland Park, Beijing. Soil samples were collected from the area surrounding Plateau Lakes (IMB20201S–IMB20212S) in the Guizhou province and sediment soil samples (IMB22101S–IMB22112S) were collected from fresh water reservoirs in the Sichuan province. Finally, water samples (IMB19301W–IMB19312W) were collected from Erhai Lake and phycosphere samples (IMB14101E–IMB14112E) were collected from the phycosphere of agar cultures maintained in our laboratory. Detailed information for samples is provided in Supplementary Table 1. Soil and sand samples were collected into sterile envelopes. Water samples were filtered through 3.0 μm pore-size filters to exclude most cyanobacterial colonies and other small particles, followed by filtering water to concentrate biomass on 0.22 μm pore-size filters. Samples were returned to the laboratory within 3 days of collection, with microbial isolation and DNA extraction carrying out immediately.

### 2.2 DNA extraction and 16S rRNA gene amplicon sequencing

Samples from the same biotopes were pooled into a single composite sample and subjected to DNA extraction for community compositional analyses. Total genomic DNA from each soil composite sample was extracted with a PowerSoil DNA isolation kit (MoBio, USA). The DNA from each composite water sample was extracted with a PowerWater DNA isolation kit (MoBio, USA) according to the manufacturer’s protocols. Total DNA was then used as template for PCR amplification of the V3 to V4 hypervariable regions of 16S rRNA genes using the universal bacterial primers 5′-ACTCCTACGGGAGGCAGCAG-3′ (338F) and 5′-GGACTACHVGGGTWTCTAAT-3′ (806R). PCR amplifications were performed using high fidelity TransStart Fastpfu DNA Polymerase (Transgen, China) in 20 μL reaction mixtures containing 4 μL of 5 × FastPfu Buffer, 2 μL of 2.5 mmol/L dNTPs, 0.8 μL of each primer (5 μmol/L), 0.4 μL of FastPfu Polymerase (final concentration was 1 unit), and 10 ng of template DNA. PCR conditions comprised 5 min of an initial denaturation at 94°C followed by 35 cycles of denaturation at 94°C for 30 s, 45 s of primer annealing at 55°C, 40 s of elongation at 72°C, and then a final 10 min elongation step at 72°C.

Purified PCR amplicons were pooled in equimolar amounts and paired-end sequenced on the Illumina MiSeq PE300 platform (Illumina, San Diego, USA) using standard protocols at Majorbio Bio-Pharm Technology Co., Ltd. (Shanghai, China). Raw FASTQ files were de-multiplexed using an in-house perl script and then quality-filtered and merged using the following criteria: (i) 300 bp reads were truncated at any site with an average quality score of < 20 over a 50 bp sliding window, and truncated reads < 50 bp were discarded, in addition to reads containing ambiguous characters; (ii) only overlapping sequences > 10 bp were merged. The maximum mismatch ratio of the overlapping region was set to 0.2 and reads that could not be assembled were discarded. (iii) Samples were distinguished based on barcoded primers, sequence direction was adjusted, and exact barcode matching was specified, in addition to setting 2 nucleotide mismatches as the maximum for primer matching. The quality-filtered sequences were clustered into operational taxonomic units (OTUs) at the 97% nucleotide sequence similarity level. The taxonomic classification of each OTU representative sequence was analyzed using the RDP Classifier (version 2.2), with comparison against the Silva 16S rRNA gene database (version 138) using a classification confidence threshold of 0.7. We carried out the high throughput sequencing analysis using Majorbio online analysis platform.^2^ The alpha diversity traits of each composite sample estimated by the Chao1 estimator, and the Shannon diversity index, in addition to the coverage estimated by Good’s coverage. Rarefaction analyses was used to show whether the amount of sequencing data in these composite samples is reasonable. Relative abundance referred to the proportion of reads of a particular taxon (OTU) in all reads of a composite sample.

### 2.3 Isolation of microorganisms

The dilution plating method was used to isolate strains from each sample using previously described procedures (Deng et al., 2022). Strain CPCC 204708*^T^* was obtained from soils of the Badain Jaran Desert (39°21′ N, 102°19′ E, 1,550 m), using media containing (L^–1^): 2.0 g sodium malonate, 0.1 g NH_4_NO_3_, 0.1 g KCl, 0.05 g MgSO_4_⋅7H_2_O, 0.05 g FeSO_4_⋅7H_2_O, 0.38 g marine trace salt mixture, 15 g agar, and pH adjustment to 7.0–7.2. Aztreonam and potassium dichromate were added to the isolation medium to final concentrations of 25 and 50 mg L^–1^, respectively, to prevent fungal and Gram-negative bacterial growth. Distinct colonies were chosen for subsequent streaking on R2A agar (Difco) to identify isolated and uniform colonies. Pure cultures were cultivated and maintained on R2A medium at 4°C and stored in aqueous glycerol suspensions (20%, v/v) at −80°C.

The reference strains *S. phytolaccae* KCTC 29190*^T^* and *S. taibaiensis* KCTC 29222*^T^* were obtained from the Korean Collection for Type Cultures (KCTC), while *S. pauli* JCM 13025*^T^* was obtained from the RIKEN BioResource Research Center (JCM). *S. ginsenosidimutans* DSM 21036*^T^* and *S. soli* DSM 22325*^T^* were obtained from the DSMZ. The reference strains were included in subsequent assays in parallel with newly identified strains.

### 2.4 Identification of *Solirubrobacter* strains

The whole genome of *Solirubrobacter* sp. URHD0082, which was isolated from Mediterranean grassland soil, was retrieved from the NCBI database (accession: AUEK00000000^3^), and was included in the pan-genomic analysis of the genus *Solirubrobacter*. The 16S rRNA gene sequence extracted from the genome was used to identify *Solirubrobacter* sp. URHD0082 based on sequence comparisons and phylogenetic analyses. In addition, genomic comparisons were used to identify the taxon to which strain URHD0082 belonged.

The genomic DNA of new isolates was extracted and their 16S rRNA genes were PCR amplified, as previously described (Li et al., 2007). The sequences were then compared against those in GenBank using the BLAST program and the EzBioCloud^4^ platform (Yoon et al., 2017) to determine approximate phylogenetic affiliations. Multiple sequence alignments of isolate and closely related 16S rRNA genes were conducted using the molecular evolutionary genetics analysis (MEGA) software package (version 7.0) (Kumar et al., 2016). Phylogenetic trees were inferred using Neighbor-Joining (Saitou and Nei, 1987), Maximum Parsimony (Kluge and Farris, 1969), and Maximum-Likelihood (Felsenstein, 1981) methods. Phylogenetic reconstruction topologies were evaluated with the bootstrap resampling method (Felsenstein, 1985) and 1,000 replicates. A phylogenetic tree was also conducted based on the concatenation of 120 ubiquitous single-copy maker genes (bac120 maker set) (Parks et al., 2018) by a pipeline called EasyCGTree (Xue et al., 2021). Average nucleotide identity (ANI) and digital DNA-DNA hybridization (dDDH) values among the strains CPCC 204708*^T^*, URHD0082 and other validly described *Solirubrobacter* species were calculated using the ezbiocloud platform (Yoon et al., 2017) and the Genome-to-Genome Distance Calculator (GGDC, version 3.0)^5^ (Auch et al., 2010), respectively.

### 2.5 Growth conditions, morphological characteristics, and physiological tests

Physiological characteristics of strain CPCC 204708*^T^* were examined by observing growth at 28°C for 1–4 weeks on peptone yeast glucose (PYG; 3% trypticase soy broth, 0.3% yeast extract, 1.5% agar; pH 7.0–7.2), tryptone soy agar (TSA, Difco), ISP 2, yeast extract sucrose (YM, Difco), Luria-Bertani, R2A (Difco), ISP 4, and nutrient (Shirling and Gottlieb, 1966) media formulations. Growth temperature ranges were evaluated by incubation on R2A agar medium at 0, 4, 10, 20, 25, 28, 30, 32, 37, and 40°C for 14 d (Sun et al., 2017). The growth pH range was measured using a previously described buffer system (Xu et al., 2005), R2A as the basal medium, and evaluation over a pH range of 4.0–11.0 (at intervals of 1 pH unit). Salt (sodium chloride) tolerance was evaluated on R2A agar medium supplemented with NaCl concentrations of 0, 1, 2, 3, 4, 5, 6, 7, 8, 9, 10, and 15% (w/v). UV radiation tolerance was evaluated using a UV-C radiation wavelength of 254 nm, following previously described procedures (Zhang et al., 2007). Growth after 10 days was determined to be either positive or negative for UV resistance compared to unirradiated controls. The strain *Deinococcus radiodurans* ATCC 13939*^T^* was also used as a positive control. The strain *Escherichia coli* ATCC 25922 was used as a negative control.

Gram stains were conducted as previously described (Magee et al., 1975). Colony appearance and pigment production were evaluated after incubation at 28°C on R2A medium. Cellular morphological features were observed with 5–7-day-old cultures using light microscopy (Zeiss Axio Scope. A1 Vario) and transmission electron microscopy (JEOL JEM-1010). Cell motility was evaluated with inverted microscopic observations of cells suspended in a 0.85% NaCl solution. Oxidase activity was evaluated using an analytical profile index (API) oxidase reagent (bioMeriéux) according to the manufacturer’s instructions, while catalase activity was determined by the presence of bubble production after application of 3% (v/v) H_2_O_2_. Metabolic characteristics were determined using Biolog GEN III (MicroPlate), API 50CH, and API ZYM (bioMerieux) test kits according to the manufacturer’s instructions. The abilities of the strain to hydrolyze gelatin, cellulose, and starch, in addition to producing H_2_S and indole were evaluated, as previously described (Gonzalez et al., 1978). The type strain *S. phytolaccae* KCTC 29190*^T^* was included in the physiological and biochemical tests for comparison.

The ability of the strains to produce indole-3-acetic acid (IAA) was evaluated with colorimetric methods (Bric et al., 1991). Briefly, log-phase cells were inoculated into 1% tryptone aqueous solutions containing 3 mmol/L L-tryptophan and then cultured at 28°C for 4 days. Absorbance at 530 nm was then plotted against IAA standard solution concentration solutions (0, 0.625, 1.0, 1.25, 2.0, 2.5, 4.0, and 5.0 mg/L), followed by linear regression to obtain a standard curve for IAA (Supplementary Figure 1). IAA quantification was based on a linear regression equation (*y* = 0.02x-0.001) for the colorimetric IAA content assay that exhibited a good fit (*R*^2^ = 0.9994), enabling quantification.

### 2.6 Chemotaxonomic assays

Chemotaxonomic and molecular systematic studies of strain CPCC 204708*^T^* were conducted with cells after cultivation in TSB medium at 28°C for 7 days in shake flasks on a rotary shaker (150 r/min) until cells reached the logarithmic growth phase. Amino acids and peptides in whole cell hydrolysates were analyzed by two-dimensional ascending thin-layer chromatography (TLC) on cellulose plates using solvent systems described by Schleifer and Kandler (1973). Sugar profiles were evaluated with TLC, as previously described (Komagata and Suzuki, 1988). Polar lipids were extracted, as previously described and identified by two-dimensional TLC (Minnikin et al., 1984). Menaquinones were extracted as previously described (Collins et al., 1977) and analyzed by HPLC (Groth et al., 1997). Cellular fatty acids analysis was performed using the Microbial Identification System (Sherlock Version 6.0; MIDI database: ACTIN1) (Kroppenstedt, 1985; Sasser, 1990).

### 2.7 Whole genome sequencing and comparative genomics

#### 2.7.1 Genome sequencing and assembly

Whole-genome sequencing of the new isolate CPCC 204708*^T^* and the reference strains *S. phytolaccae* KCTC 29190*^T^*, *S. taibaiensis* KCTC 29222*^T^*, *S. ginsenosidimutans* DSM 21036*^T^* were conducted on the Illumina HiSeq 4000 platform (Illumina, San Diego, CA, USA) at the Beijing Genomics Institute (Beijing, China) in this study. Genomic DNA was randomly sheared to construct three read libraries of length 300 bp using a Bioruptor ultrasonicator (Diagenode, Denville, NJ, USA) and physico-chemical methods. Paired-end fragment libraries were then sequenced using manufacturer protocols. Low quality reads (those with consecutive bases covered by fewer than five reads) were discarded and the remaining reads were assembled with the SOAPdenovo v1.05 software program (Xie Y. et al., 2014). The assembled genomes of strains *S. pauli* JCM 13025*^T^* and *S. soli* DSM 22325*^T^* were downloaded from NCBI database. The quality (index: completeness and contamination) of the draft genomes of the genus *Solirubrobacter* were accurately assessed by the CheckM pipeline (Parks et al., 2015).

#### 2.7.2 Genome prediction, annotation and analysis of functional genes and biosynthetic gene clusters

The assembled genomes of strains CPCC 204708*^T^*, *S. phytolaccae* KCTC 29190*^T^*, *S. pauli* JCM 13025*^T^*, *S. taibaiensis* KCTC 29222*^T^*, *S. ginsenosidimutans* DSM 21036*^T^*, *S. soli* DSM 22325*^T^*, and URHD0082 were subjected to gene prediction using Hidden Markov models in the glimmer3 software program^6^ (Delcher et al., 2007), followed by functional annotation through comparison against the Kyoto Encyclopedia of Genes and Genomes (KEGG) database^7^ (Moriya et al., 2007). Functional genes related to stress response were identified in these genomes by comparison to the Uniprot^8^ (UniProt Consortium, 2023) and Interpro^9^ databases (Paysan-Lafosse et al., 2023). Further, biosynthetic gene clusters (BGCs) were detected and characterized using the antibiotics and secondary metabolite analysis shell platform (antiSMASH; version 6.0)^10^ (Blin et al., 2021).

#### 2.7.3 Pan-genome analysis of the genus *Solirubrobacter*

The bacterial pan-genome analysis (BPGA) pipeline (version 1.3) was used to analyze *Solirubrobacter* genomic diversity and characteristics. Protein sequences used for pan-genomic analysis were annotated with the Rapid Annotation using Subsystem Technology (RAST) server (version 2.0).^11^ BPGA was conducted with default settings, as previously described (Chaudhari et al., 2016). Proteins encoded by the seven genomes were used to generate orthologous gene/protein clusters (homologous families) using the USEARCH clustering tool and then to construct phylogenetic trees using concatenations of core genes to generate a pan-matrix in BPGA. Each homolog family was assigned a homologous gene family conservation value (HGFCV) based on its frequency in the three genomes. Different conservation values (CVs) reflect the distribution frequency of the gene homologs among the 7 strains, wherein higher CVs indicate a more widely conserved gene in the 7 *Solirubrobacter* strains. Gene families with HGFCVs of 7 were considered components of the core genome, while those with values of 2–6 or 1 were considered accessory or unique genes, respectively. Core, accessory, unique, and exclusively absent genes were retrieved from the genomes using the USEARCH clustering tool. BPGA was then used for evolutionary analysis based on concatenated core gene alignments and the binary pan-matrix. The gene matrix was calculated using shared gene value presence or absence within the orthologous gene clusters. The core genome phylogenetic tree was constructed in BPGA by first extracting the protein sequences (excluding paralogs) from 20 random orthologous gene clusters. MUSCLE was then used to generate multiple sequence alignments for each gene cluster. The alignments were concatenated and a Neighbor-Joining phylogenetic tree was constructed from the concatenated matrix.

## 3 Results, analysis, and discussion

### 3.1 The distribution of *Solirubrobacter*

**FIGURE 1:**
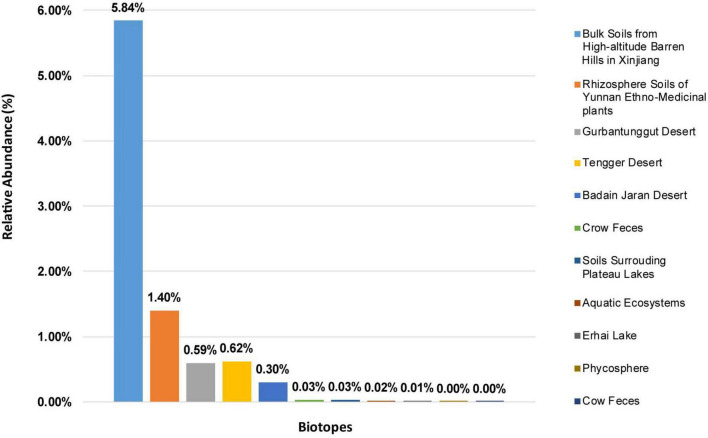
The relative abundances of *Solirubrobacter* among communities from eleven different biotopes, based on 16S rRNA gene analyses. The relative abundances are shown for composite samples from various environments, as indicated by the legend on the right.

*Solirubrobacter* spp. were detected in ecosystems based on high-throughput sequencing of 16S rRNA genes recovered from various environments. The alpha diversity of these composite samples from the different ecosystems exhibited different Chao1 and Shannon index values, suggesting significant difference in the richness or diversity of bacteria among these biotopes. Rarefaction analyses using the Shannon index as a diversity metric indicated that our sequencing efforts covered nearly all of the diversity that would be expected to be found in these composite samples. The highest abundances of *Solirubrobacter* spp. were observed in rhizosphere soils attached to medicinal plants in high-altitude areas of Xinjiang and Yunnan. They were also frequently observed in desert sandy soil samples. Low *Solirubrobacter* richness was observed in other ecosystems, including in crow feces and aquatic habitats (Figure 1). Further, *Solirubrobacter* spp. were not detected at all in the phycosphere of laboratory culture-systems, nor from cow feces collected from a Beijing farm. These results suggest that *Solirubrobacter* spp. may be adapted to life within extreme ecosystems like the high altitude barren hills of Xinjiang and Yunnan that exhibit strong solar radiation, in addition to arid deserts.

### 3.2 Identification of novel *Solirubrobacter* strains

Strain CPCC 204708*^T^* was isolated from a desert sandy soil sample (IMB16109D) collected from the Badain Jaran Desert (39°56′12′′ N, 102°05′39′′ E; 1,157 mH) from within the Inner Mongolia autonomous region. An almost complete 16S rRNA gene sequence (1,500 bp) of strain CPCC 204708*^T^* was obtained, and comparison against available sequences in GenBank revealed highest 16S rRNA gene sequence similarity to that of *Solirubrobacter phytolaccae* KCTC 29190*^T^* (98.3% nucleotide identity). The 16S rRNA gene sequence (1,427 bp) from the genome of *Solirubrobacter* sp. URHD0082 (AUEK00000000) was also extracted and compared against the Genbank database, revealing highest 16S rRNA gene sequence to that of *S. ginsenosidimutans* DSM 21036*^T^* (97.4% nucleotide similarity).

The draft genome sequences of strains CPCC 204708*^T^*, *S. phytolaccae* KCTC 29190*^T^*, *S. taibaiensis* KCTC 29222*^T^* and *S. ginsenosidimutans* DSM 21036*^T^*, were deposited in NCBI under the accessions JAPCID000000000, JAPDDP000000000, JAPDDQ000000000, and JAPDOD000000000, respectively (Table 1). The whole genome shotgun project accession number for strain URHD0082 is AUEK00000000. The GenBank accession numbers for the 16S rRNA gene sequences of strains CPCC 204708*^T^* and URHD0082 are MH509728 and OQ674416, respectively.

**TABLE 1:** Characteristics and distribution of core, accessory, unique, and exclusively absent genes of seven genomes included in the pan-genome of the genus *Solirubrobacter.*

Strain	Accession number	GenBank assembly accession	Total genes	Size (Mbp)	Isolation environment	Completeness (%)	Contamination (%)	No. of core genes	No. of accessory genes	No. of unique genes	No. of exclusively absent genes
*Solirubrobacter deserti* sp. nov. CPCC 204708^T^	JAPCID000000000	GCA_027587175.1	6,863	6.9	Sand from the Badain Jaran Desert, China	98.7	0.4	2,474	2,919	1,100	80
*Solirubrobacter ginsenosidimutans* DSM 21036^T^	JAPDOD000000000	GCA_027587205.1	8,947	9.4	Ginseng field soil from Baekdu Mountain, China.	99.6	4.4	2,474	3,767	2,099	56
*Solirubrobacter pauli* JCM 13025^T^	RBIL00000000	GCA_003633755.1	6,707	7.1	Burrow of the epigeic earthworm *Lumbricus rubellus* in an agricultural soil, USA	98.7	0.9	2,474	3,087	843	39
*Solirubrobacter phytolaccae* KCTC 29190^T^	JAPDDP000000000	GCA_027587195.1	7,265	7.5	The surface-sterilized roots of *Phytolacca acinosa* Roxb. collected from Taibai Mountain in Shaanxi Province, north-west China.	98.7	3.5	2,474	3,302	1,032	25
*Solirubrobacter soli* DSM 22325^T^	AUIK00000000	GCA_000423665.1	8,699	9.3	Soil of a ginseng field in South Korea	98.7	1.7	2,474	3,749	1,914	53
*Solirubrobacter taibaiensis* KCTC 29222^T^	JAPDDQ000000000	GCA_027587225.1	8,554	8.3	Surface-sterilized stem of *Phytolacca acinosa* Roxb. collected from Taibai Mountain in Shaanxi Province, north-west China.	98.7	7.8	2,474	3,447	1,351	27
“*Candidatus* Solirubrobacter pratensis” sp. nov. URHD0082	AUEK00000000	GCA_000425945.1	6,557	6.6	Mediterranean Grassland Soil	99.1	1.2	2,474	1,487	2,436	637

**FIGURE 2:**
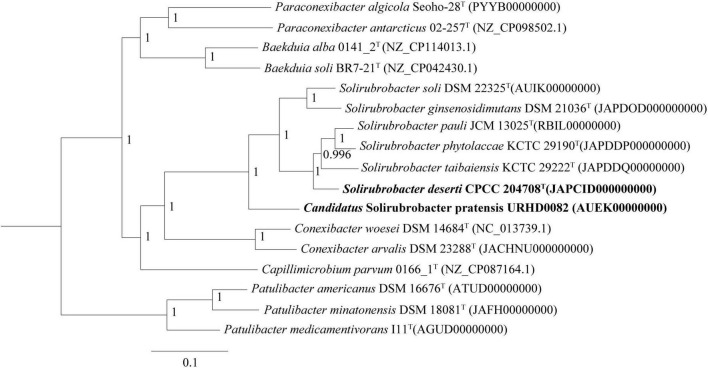
Core gene tree based on 120 ubiquitous single-copy maker genes (bac120 maker set) from whole genome sequences showing the relationship of strain CPCC 204708^T^ and URHD0082 with other species in the genus *Solirubrobacter* and other related species in the order *Solirubrobacterales*. Bootstrap values (those above 50%) are shown as percentages of 1,000 replicates. *Micrococcus luteus* ATCC 4698^T^ (GenBank accession no. QVMY00000000) was used as an outgroup (not shown). Bar, 0.1 nt substitution per nt.

Phylogenetic analysis of 16S rRNA gene sequences revealed that strains CPCC 204708*^T^* and URHD0082 formed a distinct group with the five *Solirubrobacter* species, regardless of phylogenetic reconstruction method (Supplementary Figure 2), as confirmed by the pan-matrix constructed from BPGA (Supplementary Figure 3). Thus, strains CPCC 204708*^T^* and URHD0082 were both phylogenetically affiliated to *Solirubrobacter.* In the core gene tree based on 120 ubiquitous single-copy maker genes (bac120 maker set) from whole genome sequences, strains CPCC 204708*^T^* and URHD0082 occupied distinct species positions in the genus *Solirubrobacter* (Figure 2), which was supported by the phylogenetic tree based on the 16S rRNA gene sequences (Supplementary Figure 2). The ANI values between strain CPCC 204708*^T^*, URHD0082, and the other validly described *Solirubrobacter* species were in the range of 77.3–84.4%, all being far lower than the threshold used for bacterial species delineation (ANI < 95%) (Kim et al., 2014). Further, the corresponding dDDH values ranged from 20.5 to 27.6% (Supplementary Table 2), which were also far below the threshold value (70%) used to identify bacterial strains of the same species (Auch et al., 2010). Based on these analyses, we proposed that the two strains identified here represent novel *Solirubrobacter* species, for which the epithets *Solirubrobacter deserti* sp. nov. and *Candidatus* “Solirubrobacter pratensis” sp. nov. are suggested, with strains CPCC 204708*^T^* and URHD0082*^T^* as the types, respectively.

### 3.3 Morphological and physiological characteristics

Strain CPCC 204708*^T^* grew well on R2A, TSA and YM media, with moderate growth on ISP4 and PYG media, while no growth was observed on ISP 2, Luria-Bertani, and nutrient media. Strain CPCC 204708*^T^* growth was observed at 20–37°C, pH 5.0–8.0, and in presence of 0–10% (w/v) NaCl. Optimum growth occurred at 28–30°C, pH 7.0–8.0, and without NaCl addition. Strain CPCC 204708*^T^* colonies on R2A medium were circular, convex, and smooth, with pale pink color and approximately 1.9 mm in diameter. Diffusible pigments were not produced in the media. When grown on R2A medium, cells were aerobic, Gram-stain positive, non-motile, non-spore-forming, and rod-shaped, with sizes of 0.6–0.8 × 1.2–1.9 μm (Supplementary Figure 4). The cells were positive for catalase activity, but negative for oxidase activity. Detailed physiological and biochemical characteristics of strain CPCC 204708*^T^* are shown in Table 2 and in the species description.

**TABLE 2:** Differentiating characteristics between strain CPCC 204708^T^ and the type strains of other *Solirubrobacter* species.

Characteristic	1	2	3	4	5	6
Colony color	Pink	White	Pink	White	Pale yellow	White
Temperature range (°C)	20–37	15–33	15–42	7–33	25–37	15–37
pH range	5.0–8.0	6.0–9.0	6.0–7.5	6.0–10.0	6.0–7.0	6.0–10.0
NaCl Tolerance (%, w/v)	0–10	0–7^a^	0–10^a^	0–7^a^	0–3^a^	0–7^a^
Liquefaction of gelatin	–	+	–	+	–	+
Oxidase activity	–	–	–	+	+	+
Hydrolysis of starch	–	–	+	+	–	–
**Acid production from**						
N-acetylglucosamine	+	–	–	–	–	+
D-arabinose	–	+	+	–	+	+
D-glucose	–	–	+	–	–	+
D-maltose	–	–	+	–	–	+
Potassium gluconate	+	–	+	–	–	–
**Enzyme activities**						
Lipase (C 14)	+	+	+	+	–	–
β-galactosidase	–	+	–	–	+	+
N-acetyl-β-glucosaminidase	–	+	–	+	+	+
α-mannosidase	–	+	+	–	–	+
Concentration of IAA produced (mg/L)	0.77 ± 0.000	1.12 ± 0.000	3.50 ± 0.001	0.80 ± 0.002	2.22 ± 0.001	0.55 ± 0.002
Tolerance to UV-C radiation (102 J/cm^2^)	+	+	+ +	–	–	–
Major fatty acids	iso-C_16:0_, C_18:1_ω9*c*, iso-C_16:0_ 2OH	iso-C_16:0_, C_18:1_ω9*c*, iso-C_17:1_ω8*c*	iso-C_16:0_, C_18:1_ω9*c*	iso-C_16:0_, C_18:1_ω9*c*, C_17:1_ω8*c*, C_18:3_ω6*c* (6, 9, 12)	iso-C_16:0_, C_18:1_ω9*c*, C_18:3_ω6*c* (6, 9, 12), iso-C_16:0_ 3OH	iso-C_16:0_, C_18:1_ω9*c*
G + C content (%)	71.9	71.6	72.1	71.6	70.6	71.5

1, CPCC 204708^T^ (data from this study); 2, *S. phytolaccae* KCTC 29190^T^ (data from this study); 3, *S. pauli* JCM 13025^T^ (Singleton et al., 2003); 4, *S. taibaiensis* KCTC 29222^T^ (Zhang L. et al., 2014); 5, *S. ginsenosidimutans* DSM 21036^T^ (An et al., 2011); 6, *S. soli* DSM 22325^T^. + , positive (the number of “ + “ represents the degree of positivity); –, negative; w, weakly positive;

^a^data from this study.

Ultraviolet radiation tolerance experiments showed that strains CPCC 204708*^T^*, *S. phytolaccae* KCTC 29190*^T^* and *S. pauli* JCM 13025*^T^* could all survive when exposed to the dose of ultraviolet radiation (102 J/cm^2^), with survival rates following the order of *S. pauli* JCM 13025*^T^*, CPCC 204708*^T^* and *S. phytolaccae* KCTC 29190*^T^*. JCM 13025*^T^* and CPCC 204708*^T^* colony colors were both pink when grown on R2A media. Some radiation-resistant bacteria isolated from irradiated soils contain multiple pigments and are also more resistant to radiation than non-pigmented bacteria (An et al., 2011; Pulschen et al., 2015). For instance, the UV and cold tolerance of a purple violet pigment (PVP)-producing Antarctic bacterium *Janthinobacterium* sp. Ant5-2 was reported previously. Compared with the wild type *Janthinobacterium* sp. Ant5-2 PVP(+), the survival rate of mutant strain [PVP(-)] after ultraviolet irradiation (UV-B and UV-C) was significantly reduced (Mojib et al., 2013). Saxena et al. (2002) found that survival of Bt-m (an UV-resistant mutant of *Bacillus thuringiensis* subsp. *kurstaki*, producing a dark brown pigment, identified as melanin) spores and their insecticidal activity to irradiation at 254 nm and 366 nm were higher than those of the parent. Reis-Mansur et al. (2019) found that the increased survival of DNA repair-proficient *E. coli* grown overnight with added carotenoids (pigment extract) produced by *Microbacterium* sp. LEMMJ01 (isolated from Antarctic soil) revealed that part of the resistance of *Microbacterium* sp. LEMMJ01 against UV-B radiation seems to be connected with photoprotection by its pigments (carotenoids). Consequently, the pigment of strains JCM 13025*^T^* and CPCC 204708*^T^* may contribute greatly to their higher survival under UV radiation.

**FIGURE 3:**
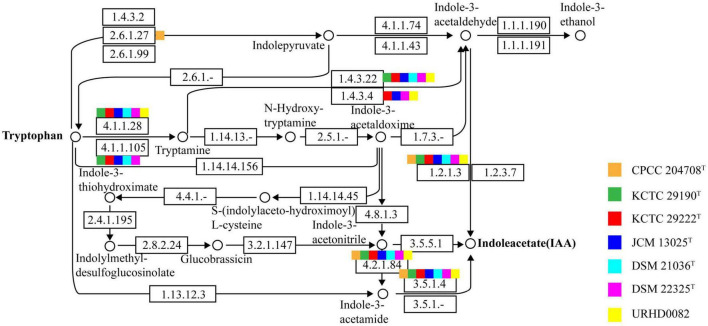
The indole-3-acetic acid (IAA) production pathway of *Solirubrobacter* species. Squares in orange, green, red, blue, light blue, pink, and yellow correspond to strains CPCC 204708^T^, KCTC 29190^T^, KCTC 29222^T^, JCM 13025^T^, DSM 21036^T^, DSM 22325^T^, and URHD0082, respectively.

Indole-3-acetic acid is an important phytohormone that benefits plant growth and development. Many bacteria produce IAA, which when provided in an optimal concentration range, can stimulate plant root hair formation and increase the numbers and lengths of lateral roots and taproots (Davies, 1995). A positive correlation between the genus *Solirubrobacter* and plant growth was also observed (Franke-Whittle et al., 2015). Subsequently, the genus *Solirubrobacter* was detected as a dominant group in soils (Sánchez-Marañón et al., 2017), rhizosphere habitats of various crops and medicinal plants (Aguiar et al., 2020; Barajas et al., 2020; Feng et al., 2021; Lee et al., 2021), and was recognized as a kind of plant probiotic (Li et al., 2022). Several multifunctional rhizosphere soil microorganisms including *Bacillus*, *Solirubrobacter*, and *Lysobacter* with higher abundance in commercial organic fertilizer plus bioorganic fertilizer (CBF) were shown to promote plant growth (Franke-Whittle et al., 2015) by producing hormones such as IAA (indole-3-acetic acid), gibberellin, and cytokinin (Fabra et al., 2010; Mhatre et al., 2018). Our phenotypic experiments demonstrated that six strains of the genus *Solirubrobacter* could produce IAA. IAA was detected in the fermentation broths of strains CPCC 204708*^T^*, *S. phytolaccae* KCTC 29190*^T^*, *S. taibaiensis* KCTC 29222*^T^*, *S. pauli* JCM 13025*^T^*, *S. ginsenosidimutans* DSM 21036*^T^*, and *S. soli* DSM 22325*^T^* at concentrations of 0.77 ± 0.000, 1.12 ± 0.001, 0.80 ± 0.002, 3.50 ± 0.001, 2.22 ± 0.001, and 0.55 ± 0.002 mg/L, respectively (Supplementary Figure 1). Production of phytohormones (such as IAA) of plant endophytes (Crozier et al., 1988) stimulate growth and/or ameliorate the plant under harsh stressful conditions (Cohen et al., 2009; Piccoli et al., 2011). Given that all reported strains of the genus *Solirubrobacter* so far have been isolated from soils or the ecosystems that were vegetated or associated with crops, especially medicinal plants, we infer that the production of small amounts of IAA could represent an important mechanism of plant-microbe interaction for this genus (Duca et al., 2014). In addition, at the genomic level, we found that the IAA-production pathway was encoded by the seven *Solirubrobacter* strains. Specifically, the genes for aldehyde dehydrogenases (EC 1.2.1.3; *aldH*) and amidases (EC 3.5.1.4; *amiE*) were both present within the core genome of *Solirubrobacter* strains. Combining phenotypic and genotypic characteristics, the complete IAA production pathway (Figure 3) within the broader tryptophan metabolism pathways (Supplementary Figure 5) were identified for the strains based on annotation against the KEGG database.

### 3.4 Chemotaxonomic characteristics

Strain CPCC 204708*^T^* showed chemotaxonomic features consistent with the genus *Solirubrobacter*. In the whole cell hydrolysates of strain CPCC 204708*^T^*, *meso*-diaminopimelic acid was detected as the signature amino acid, and galactose, xylose, rhamnose, and ribose were the components of the sugar profile. Diphosphatidylglycerol (DPG), phosphatidylglycerol (PG), phosphatidylinositol (PI), phosphatidylinositol mannosides (PIM), an unidentified aminophospholipid (APL), and an unidentified phospholipid (PL) were the primary components of the polar lipid profile (Supplementary Figure 6). The predominant menaquinone of cells was MK-7(H_4_), which is consistent with other species of the genus *Solirubrobacter* (Kim et al., 2007; An et al., 2011; Wei et al., 2014; Zhang L. et al., 2014). The major cellular fatty acids (>10%) were iso-C_16:0_, C_18:1_ω9*c*, and iso-C_16:0_ 2-OH, and the detailed composition is provided in the species description and in Supplementary Table 3. It was obvious that strain CPCC 204708*^T^* shared the major fatty acids of iso-C_16:0_ and C_18:1_ω9*c* with other validly described *Solirubrobacter* species, while the detailed fatty acids profiles could differentiate them from each other (Table 2; Supplementary Table 3). Overall, the chemotaxonomic analyses supported the classification of strain CPCC 204708*^T^* as a new member of the genus *Solirubrobacter*, consistent with the 16S rRNA gene sequence and phylogenetic analyses.

### 3.5 Genomic properties

The genomic DNA G + C content of strain CPCC 204708*^T^* was 71.9% based on its draft genome sequence. Further detailed genomic characteristics for the seven strains were summarized in Table 1 and Supplementary Appendix 1.

**FIGURE 4:**
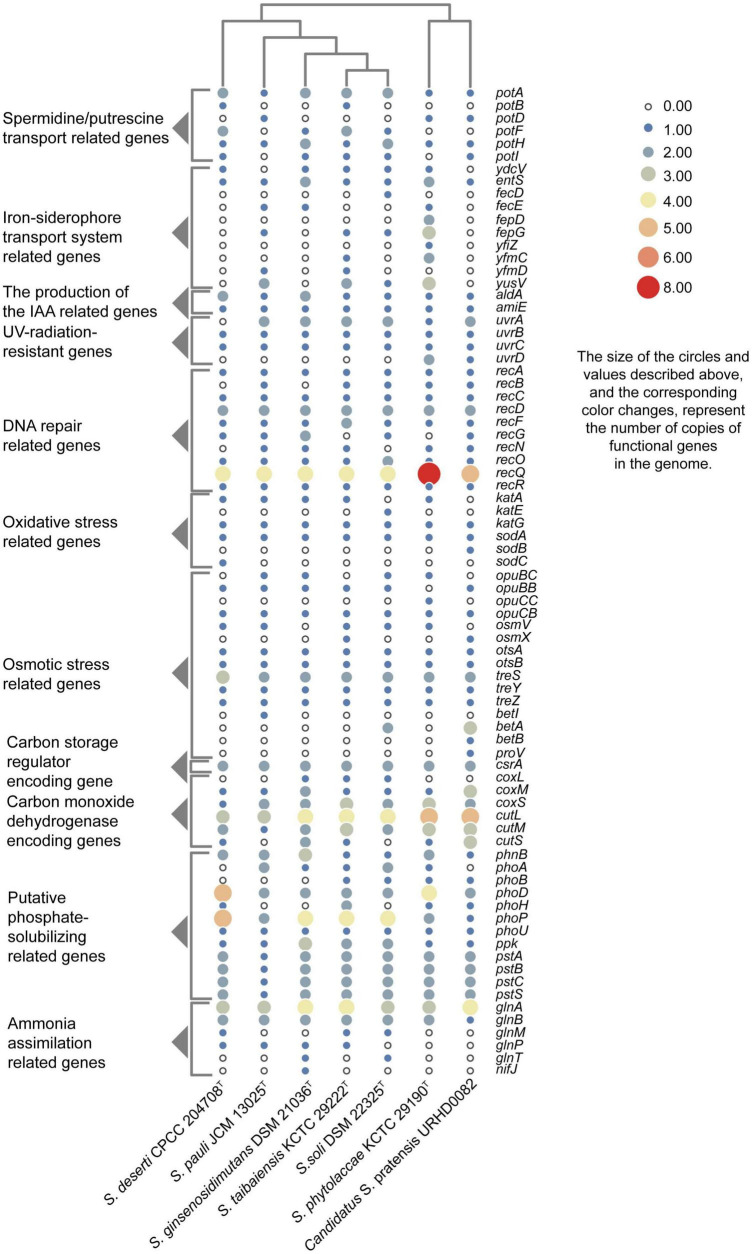
Heatmap showing the copy numbers of functional genes predicted in the genomes of seven *Solirubrobacter* species. Functional genes were identified based on comparison to the KEGG database.

Putative genes encoding catalase (*katG*) and superoxide dismutase (*sodA*) were identified in all genomes that likely help mitigate oxidative stress. In addition, the UvrABC repair system (Truglio et al., 2006) and other DNA recombination and repair-related genes were identified. As well, genes were identified that were associated with polyamine transport, osmoprotectant capacity, carotenoid biosynthesis, IAA production, iron-siderophore transport system, carbon monoxide dehydrogenase, carbon storage regulation, nitrogen assimilation, and phosphate-transport and solubilization (Figure 4).

Besides lack of precipitation in such harsh ecosystems, heterotrophic microorganisms have to challenge extreme starvation for carbon and other energy substrates. Diverseand viable microbial communities are present in the sandy soils of most deserts. To explore the potential of the desert-derived strains of this study to assimilate carbon and other energy substrates, genes associated with these capacities were annotated and identified. Carbon monoxide dehydrogenase encoding genes (*coxL*, *coxM*, *coxS, cutL, cutM*, and *cutS*) and a carbon storage regulation coding gene (*csrA*) were retrieved from the genomes (Figure 4). In addition, several genes related to acquisition and assimilation of phosphorus were identified, including *phnB*, *phoA*, *phoB*, *phoD*, *phoH*, *phoP*, *phoU*, *ppk*, *pstA*, *pstB*, *pstC*, and *pstS* (Figure 4). Consistently, phenotypic experiments indicated that strain CPCC 204708*^T^* encoded acid phosphatase and alkaline phosphatase. Nitrogen assimilation genes were also identified in the genomes of the seven strains (Figure 4). Specifically, genes encoding glutamine synthetase (*glnA*), the nitrogen regulatory protein P-II 1 (*glnB*), a probable glutamine ABC transporter permease protein (*glnM* and *glnP*), and the probable sodium/glutamine symporter GlnT (*glnT*) were identified. Glutamine synthetase encoded by *glnA* is a key multitasking protein involved in ammonium assimilation and in the regulation of genes involved in nitrogen metabolism (Schumacher et al., 2015). GlnA may bethat *glnA* is involved in ammonia assimilation under ammonia-starvation conditions, while P-II indirectly controls the transcription of *glnA*. The proteins encoded by *glnM* and *glnP* form components of the ABC transporter complex GlnHMPQ involved in glutamine transport (Yoshida et al., 2003). Moreover, plants have evolved sophisticated mechanisms to mitigate stress from fluctuating nitrate levels and can recruit microorganisms to improve nitrogen uptake (Chai et al., 2022). The bacteria associated with nitrogen transformation, such as *Solirubrobacter* spp., etc., were highly abundant; these bacteria may possess the ability to increase nitrogen availability in the crude oil-contaminated soil (Gao et al., 2022). Nitrogen fixation related gene *nifJ* was only retrieved from the genome of the strain *S. ginsenosidimutans* DSM 21036*^T^*. Thus, these *Solirubrobacter* strains may potentially promote nitrogen absorption by their symbiotics in the desert environments, thereby potentially improving their growth in these niches. Consequently, these genomic observations highlight the potential important contributions of the proteins toward adaptation to carbon, nitrogen, and energy starvation in *Solirubrobacter*.

ATP-binding cassette (ABC) transporters are one of the largest known protein families, and are ubiquitous among bacteria. The transporters couple ATP hydrolysis to active transport of diverse substrates including ions, sugars, lipids, sterols, peptides, proteins, and drugs. To explore the transport potential of the seven strains, we analyzed the related genome sequences with KEGG database and blasted with TCDB^12^ (Supplementary Appendix 2), and genes encoding ABC transporters were identified (Supplementary Figure 7). Annotation against the KEGG database revealed the presence of spermidine/putrescine transport related genes (*potA*, *potB*, *potC*, and *potD*) in the seven *Solirubrobacter* genomes (Figure 4). Higher polyamine levels in plants can minimize harmful effects resultant from biotic and abiotic stresses (salt, drought, UV, temperature, heavy metals, etc.) (Takahashi and Kakehi, 2010; Hussain et al., 2011; Shi and Chan, 2014). Inoculation with beneficial rhizobacterium *Pseudomonas putida* GAP-P45 led to increased levels of the expression of most polyamine biosynthetic genes and cellular polyamine levels in *Arabidopsis thaliana*, resulting in resistance to water-stressed conditions (Sen et al., 2018). Rhizobacteria also could modulate the redox state of salinity-affected plants by enhancing polyamines and antioxidants, which leads to increased photosynthetic efficiency (Radhakrishnan and Baek, 2017). Given that all reported strains of the genus *Solirubrobacter* so far were isolated from soils or the ecosystems that were vegetated or associated with crops, especially medicinal plants, and that polyamine transport-related genes were retrieved from the genomes of all seven strains of the genus, we concluded that *Solirubrobacter* spp. might have the potential or relate to the growth promotion of plants through the transport of polyamines. Other complete transport-related genes were identified in the seven genomes of the genus *Solirubrobacter*, including those related to transport of osmoprotectants, raffinose/stachyose/melibiose (*msmE*, *msmF*, *msmG* and *msmK*), nucleosides (*bmpA*, *nupB*, *nupC*, and *nupA*), D-xylose (*xylF*, *xylH*, and *xylG*), erythritol (*eryG*, *eryF*, and *eryE*), phosphate, branched-chain amino acids (*livK*, *livH*, *livG*, *livM*, and *livF*), D-methionine (*metQ*, *metI*, and *metN*), iron-siderophores, and lipo-oligosaccharides (*nodJ* and *nodI*).

Salt tolerance assays revealed that strains CPCC 204708*^T^*, *S. phytolaccae* KCTC 29190*^T^*, *S. taibaiensis* KCTC 29222*^T^*, *S. pauli* JCM 13025*^T^*, *S. ginsenosidimutans* DSM 21036*^T^*, and *S. soli* DSM 22325*^T^* grew in the presence of 0–10, 0–7, 0–7, 0–10, 0–3, and 0–7% NaCl (w/v), respectively. Thus, most *Solirubrobacter* species were able to tolerate certain levels of osmotic pressure. Genomic analysis revealed the presence of several genes related to osmoprotectant capacity, including genes encoding the choline transport system permease protein OpuBB (*opuBB*), choline ABC transporter substrate-binding lipoprotein OpuBC (*opuBC*), glycine betaine/carnitine/choline transport system permease protein OpuCB (*opuCB*), glycine betaine/carnitine/choline-binding protein OpuCC (*opuCC*), osmoprotectant import ATP-binding protein OsmV (*osmV*), osmoprotectant-binding protein OsmX (*osmX*), trehalose-6-phosphate synthase (*otsA*), trehalose-phosphate phosphatase (*otsB*), malto-oligosyltrehalose trehalohydrolase (*treZ*), trehalose synthase/amylase TreS (*treS*), maltooligosyl trehalose synthase (*treY*), HTH-type transcriptional regulator BetI (*betI*), oxygen-dependent choline dehydrogenase (*betA*), betaine aldehyde dehydrogenase (*betB*), and glycine betaine/proline betaine transport system ATP-binding protein ProV (*proV*) (Figure 4). Notably, many of these proteins are involved in trehalose uptake and associated biosynthesis pathways. Trehalose is a major compatible solute involved in osmotic stress responses of cells, cellular adaptation, and survival under heat and desiccation stress (Reina-Bueno et al., 2012). Lee et al. (2021) found that in high-salinity rhizosphere soil habitats planted with tomato, the abundance of some actinobacteria (such as *Solirubrobacter*) increased and the community structure tended to be stable, indicating that strains of these groups could tolerate high osmotic pressure and potentially help plants tolerate high salt environment through complex plant-microbial interaction.

### 3.6 Pan-genomic analysis of the genus *Solirubrobacter*

A total of 50,768 protein-coding genes (Table 1) were identified among the seven *Solirubrobacter* genomes that comprised 19,800 homologous families based on cluster analysis. Homologous gene family conservation values (HGFCVs) were evaluated among the homolog clusters (Supplementary Figure 8A). A total of 2,474 core genes were shared by the seven strains (HGFCV = 7), accounting for ∼12.5% of the total homologous gene families, while accessory genes (HGFCVs = 2–6) accounted for ∼33.1% of the gene families (6,551 genes) in the genus *Solirubrobacter*. In addition, unique genes (HGFCV = 1) comprised ∼54.4% of the total (10,775 genes).

The functional relationship between pan-genome size (f*_*pan*_*) and the number of genomes (n) was obtained by evaluating the following equation:


$${\text{f}}_{\text{pan}}\,\left(n\right)=7155.93\times n^{0.522211}$$

In addition, the functional relationship between the number of core genes (*f*_*core*_) and the number of genomes (n) was obtained by evaluating the following equation:


$${\text{f}}_{\text{core}}\,\left(n\right)=6920.36\times {\text{e}}^{-0.211409\,n}$$

Please refer to the reference (Chaudhari et al., 2016) for detailed derivation. With increasing numbers of sequenced genomes, the pan-genome size increased, rather than plateauing (Supplementary Figure 8B), suggesting that pan-genome size may continue to enlarge if the number of genomes of this genus continue to increase. Thus, the pan-genome of *Solirubrobacter* may be of an open type. Open pan-genomes are associated with species of the genus inhabiting multiple environments and having multiple ways of exchanging genetic material (Medini et al., 2005). Therefore, it is reasonable to infer that many unknown species of the genus *Solirubrobacter* inhabited in other biotopes have yet to be discovered.

Of the 19,800 genes (clusters), BPGA mapped 6,216 (31.4%) to KEGG database pathways, including for 2,087 core genes (33.6%), 2,017 accessory genes (32.4%), and 2,112 unique genes (34.0%). KEGG pathway results related to eukaryotes (e.g., Human Diseases and Organismal Systems) were removed in order to construct a metabolic reconstruction of the *Solirubrobacter* pan-genome. Many core genes (1,997) were involved in carbohydrate metabolism (17.0%), other carbon metabolism pathways (biosynthesis of amino acids, 5.8%; carbon metabolism, 4.9%; 2-oxocarboxylic acid metabolism, 1.6%; fatty acid metabolism, 1.3%; and degradation of aromatic compounds, 0.4%) (14.0% total), amino acid metabolism (13.7%), energy metabolism (7.6%), metabolism of cofactors and vitamins (5.8%), nucleotide metabolism (4.9%), lipid metabolism (4.4%), replication and repair (4.3%), signal transduction (4.1%), xenobiotic biodegradation and metabolism (4.0%), translation (3.8%), membrane transport (2.8%), metabolism of other amino acids (2.7%), metabolism of terpenoids and polyketides (2.5%), biosynthesis of other secondary metabolites (1.9%), cell motility (1.9%), folding, sorting, and degradation (1.8%) and glycan biosynthesis and metabolism (1.3%). Accessory and unique genes were enriched in pathways related to carbohydrate metabolism, amino acid metabolism, and the metabolism of other substrates.

Among the accessory genes (1,886), most encoded proteins related to the pathways of carbohydrate metabolism (15.0%), amino acid metabolism (14.3%), other specific carbon metabolism pathways (carbon metabolism, 3.8%; biosynthesis of amino acids, 2.6%; fatty acid metabolism, 1.7%; degradation of aromatic compounds, 0.9% and 2-oxocarboxylic acid metabolism, 0.6%) (9.8%), signal transduction (8.8%), membrane transport (7.4%), xenobiotic biodegradation and metabolism (7.3%), lipid metabolism (6.3%), energy metabolism (6.0%), metabolism of cofactors and vitamins (5.6%), metabolism of other amino acids (3.2%), nucleotide metabolism (2.9%), biosynthesis of other secondary metabolites (2.6%), metabolism of terpenoids and polyketides (2.3%), cell motility (2.2%), translation (1.3%), transport and catabolism (1.1%), and glycan biosynthesis and metabolism (1.0%).

**FIGURE 5:**
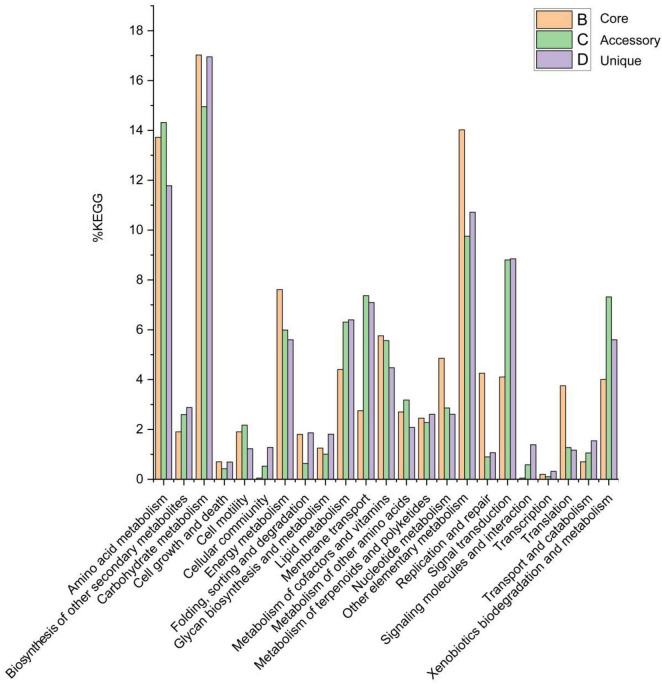
Abundances of metabolic pathways associated with core, accessory, and unique genes within the *Solirubrobacter* pan-genomic analyses. Metabolic pathways were identified from the KEGG database.

Among the 1,876 unique genes, their encoded proteins were related to pathways associated with carbohydrate metabolism (17.0%), amino acid metabolism (11.8%), other specific carbon compound metabolism (carbon metabolism, 4.3%; biosynthesis of amino acids, 2.8%; fatty acid metabolism, 2.3%; 2-oxocarboxylic acid metabolism, 0.7% and degradation of aromatic compounds, 0.6%) (10.7%), signal transduction (8.8%), membrane transport (7.1%), lipid metabolism (6.4%), energy metabolism (5.6%), xenobiotic biodegradation and metabolism (5.6%), metabolism of cofactors and vitamins (4.5%), biosynthesis of other secondary metabolites (2.9%), nucleotide metabolism (2.6%), metabolism of terpenoids and polyketides (2.6%), metabolism of other amino acids (2.1%), folding, sorting, and degradation (1.9%), glycan biosynthesis and metabolism (1.8%), transport and catabolism (1.5%), signaling molecules and interactions (1.4%), cellular community (1.3%), cellular motility (1.2%), translation (1.2%) and replication and repair (1.1%) (Figure 5).

The pan-genome of the genus *Solirubrobacter* is characterized by a high proportion of carbohydrate metabolism, amino acid metabolism, and energy metabolism, these genomic features suggest that these strains have the potential to assimilate more sources of carbon and nitrogen to cope with extreme starvation of carbon and other energy substrates. These genomic-level characteristics also suggest that members of the genus *Solirubrobacter* might play an important role in soil organic matter assimilation and biogeochemical cycling.

Compared with the core genomes of the species *Modestobacter deserti* (Jiang et al., 2021) and the genus *Geminicoccus* (Jiang et al., 2022) by BPGA in our previous studies, we found that spermidine/putrescine transport related genes (*potA*, *potB*, *potC* and *potD*) were retrieved only in the core genome of seven strains of the genus *Solirubrobacter*, with no spermidine/putrescine transport related genes in the genomes of *Modestobacter deserti* and the genus *Geminicoccus*. While spermidine/putrescine transport related genes were present in the genomes of *Conexibacter* spp., a group of actinobacteria mostly isolated from the vegetated biotopes, a small number of *Conexibacter* spp. isolated from desert and aquatic habitats had only *potA* genes, or were missing any *pot* genes (data unpublished). Integrating the information from all validly described strains of the genus *Solirubrobacter* derived from the vegetated biotopes or the ecosystems associated with crops, we supposed that spermidine/putrescine transporter encoding genes might correlate to vegetation (Leontidou et al., 2020). Leontidou et al. (2020) experimentally verified that most selected Plant Growth Promoting Rhizobacteria (PGPR) harbored the genes responsible for polyamine biosynthesis. Accordingly, we supposed that the polyamine related pathway probably act as an additional PGPR-related mechanism involved in plant growth promotion, which need to be further explored. Polyamine (e.g., spermidine) is essential for eukaryotic cell viability and is correlated with lateral root development, pathogen resistance and alleviation of oxidative, osmotic and acidic stresses (Xie S. S. et al., 2014).

Genomes are subject to damage by chemical and physical agents in environments (e.g., UV and ionizing radiation, fungal or bacterial toxins, and chemical mutagens) and by free radicals endogenously generated during cellular metabolism (Tuteja et al., 2001). A variety of different DNA repair pathways help mitigate DNA damage and enable cells to withstand the high solar radiation encountered in desert habitats. UV radiation analysis revealed that strain CPCC 204708^T^, *S. phytolaccae* KCTC 29190^T^, and *S. pauli* JCM 13025^T^ survived exposure to 254 nm ultraviolet radiation. Within the core genome of the seven strains analyzed here, genes were identified associated with the UvrABC repair system (Truglio et al., 2006; Supplementary Figure 9) (*uvrA*, *uvrB*, *uvrC*, and *uvrD*) and other DNA recombination and repair pathways (*recA*, *recB*, *recC*, *recD*, *recF*, *recG*, *recO*, and *recR*) (Taylor and Smith, 1999; Supplementary Figure 10). The DNA repair pathways related genes *uvr* and *rec* were also retrieved in the core-genomes of the genus *Geminicoccus*, the genus *Herbiconiux*, the genus *Conexibacter* and the species *Modestobacter deserti*.

β-Glucosidase activity was also observed for strain DSM 21036^T^ and this activity was demonstrated as responsible for the gradual conversion of ginsenoside Rb_1_ to the compound F_2_ (An et al., 2011). β-glucosidase activity was also observed for strain CPCC 204708^T^ and the five other *Solirubrobacter* strains. Consistently, beta-glucosidase encoding genes (*bgl*) were identified in the core genome of the *Solirubrobacter* strains. The *bgl* gene (encoding β-glucosidase) was retrieved in the core-genomes of the genus *Geminicoccus*, the genus *Herbiconiux*, and the species *Modestobacter deserti*, but not in the core-genome of the genus *Conexibacter*.

### 3.7 Secondary metabolite biosynthesis gene cluster analysis

The secondary metabolism of actinobacteria, especially actinobacteria from extreme habitats, is a rich source of novel bioactive compounds with potential medicinal value. In order to identify new drug candidates, microbiologists are increasingly combining multi-omics techniques to predict the potential for secondary metabolite synthesis by sequencing the genomes of various microorganisms. Here, we identified the secondary metabolite biosynthetic gene clusters (BGCs) of genus *Solirubrobacter* by antiSMASH.

The annotation of secondary metabolite biosynthesis gene clusters in the seven *Solirubrobacter* genomes revealed the presence within each genome of eight to thirteen secondary metabolite gene clusters, which exhibited low similarities to previously described secondary metabolite biosynthetic gene clusters. Specifically, the gene clusters exhibited 5–40% nucleotide similarities to known secondary metabolite biosynthetic gene clusters including those for accramycin A, microansamycin, calcium-dependent lipopeptide, lomofungin, tiancimycin, schizokinen, lankacidin C, linfuranone B/C, kitasetaline, fulvuthiacene A/B, macrotermycins. In addition, other unidentified secondary metabolite clusters were identified that were attributable to those encoding NAPS-independent-siderophores, lassopeptides, LAP thiopeptides, terpenes, NAPAAs, redox-cofactors, RiPP-like compounds, RRE-containing compounds, ranthipeptides, indole, and lanthipeptide-class-iv types (Supplementary Table 4). The analysis with antiSMASH also revealed the presence of a microansamycin gene cluster in strain CPCC 204708^T^. Pentaketide ansamycins have rarely been reported and include compounds like the antioxidant Q-1047, lipoxygenase inhibitor tetrapetalones (Komoda et al., 2004), radical scavenger ansaetherones (Komoda et al., 2008), cebulactams (Pimentel-Elardo et al., 2009), and the macrodilactam juanlimycins (Zhang J. et al., 2014). The novel pentaketide ansamycin, Microansamycin D, was recently identified and its antioxidant activity was confirmed (Wang et al., 2018). Accramycin A is a new naphthacene-type aromatic natural product and was identified in the antiSMASH analysis of strain CPCC 204708^T^. Accramycin A was first discovered from the metabolites of *Streptomyces* sp. MA37 (Maglangit et al., 2019). The antibacterial activities of accramycin A were also preliminarily evaluated against Group B *Streptococcus*, revealing a minimum inhibitory concentration (MIC) of 27 μg/mL, providing the first evidence of naphthacene-type aromatic polyketide bioactivity. The Lomofungin gene cluster was also identified in the antiSMASH analysis of strain *S. phytolaccae* KCTC 29190^T^. This antibiotic exhibits antibacterial activity against fungi, yeast, and bacteria (Klo et al., 1973). Further, macrotermycins A and C have exhibited antimicrobial activity against human pathogenic *Staphylococcus aureus* in addition to selective antifungal activity against a fungal parasite from termite fungal gardens (Beemelmanns et al., 2017).

The pangenomes of the genera *Geminicoccus*, *Herbiconiux* and *Conexibacter*, as well as the species *Modestobacter deserti* were used as the control genomes of the genus *Solirubrobacter*. Upon comparison, it was discovered that secondary metabolism cluster profiles in the pangenome of the genus indeed showed specific. The secondary metabolite gene clusters responsible for accramycin A, microansamycin, calcium-dependent lipopeptide, lomofungin, tiancimycin, schizokinen, lankacidin C, linfuranone B/C, kitasetaline, fulvuthiacene A/B, and macrotermycins, were only retrieved from the genomes of genus *Solirubrobacter*, but not from those of the genus *Herbiconiux*, *Geminicoccus*, *Conexibacter* (data unpublished), and the species *Modestobacter deserti*.

## 4 Conclusion

In this study, the distribution of the genus *Solirubrobacter* was evaluated across numerous environments, revealing their enrichment in soils of areas with high UV radiation (e.g., desert soils). In addition, the novel strain *Solirubrobacter deserti* sp. nov. CPCC 204708^T^ was isolated, identified, and characterized, in addition to subsequent characterization of the genetic basis of *Solirubrobacter* adaptations to harsh environments and their potential mediation of plant-microbe interactions. Further, strain URHD0082 was identified as *Candidatus* “Solirubrobacter pratensis” based on genome information. Genome-scale analysis of strain CPCC 204708^T^ revealed the molecular basis for their adaptations to desert environments via mitigation of stress from UV radiation, carbon starvation, desiccation, and osmotic stress. In the absence of macrophytic phototrophs, such as in desert soils, such microorganism could potentially serve as significant contributors to both primary productivity and biogeochemical activities, thereby assuming the role of pioneering organisms. Global analysis of *Solirubrobacter* genomes and their environmental distributions suggest they are abundant in ecosystems associated with plants, where they may promote plant health.

## 5 Description of *Solirubrobacter deserti* sp. nov. and *Candidatus* “*Solirubrobacter pratensis*” sp. nov.

*Solirubrobacter deserti* (de.ser’ti. L. gen. n. *deserti* of a desert).

Cells are aerobic, Gram-stain positive, non-motile, non-spore-forming, and rod-shaped, with sizes of 0.6–0.8 × 1.2–1.9 μm. Diffusible pigments are not produced on any media. Colonies on R2A medium are circular, convex, smooth, and entire, with pale pink coloration and diameters of approximately 1.9 mm. Growth occurs at 20–37°C (optimum: 28–30°C) and at pH 5.0–8.0 (optimum: 7.0–8.0), and up to 10% (w/v) NaCl. Cells are catalase-positive and oxidase-negative, but negative for H_2_S and indole production. Cells cannot hydrolyze cellulose, starch, Tween 40, and gelatin. API ZYM strip analysis indicated that cells are positive for enzymatic activities including acid phosphatase, alkaline phosphatase, cystine arylamidase, esterase (C4), esterase lipase (C8), N-acetyl-β-glucosaminidase, β-glucosidase, leucine arylamidase, lipase (C 14), trypsin, and valine arylamidase. Acetoacetic acid, D-cellobiose, dextrin, D-fructose, D-galactose, D-galacturonic acid, D-galacturonic acid, D-gluconic acid, D-glucose-6-PO_4_, D-glucuronic acid, D-maltose, D-mannitol, D-mannose, D-melibiose, D-saccharic acid, D-trehalose, D-turanose, gelatin, glucuronamide, glycerol, L-aspartic acid, L-fucose, L-histidine, L-rhamnose, mucic acid, N-Acetyl-D-glucosamine, pectin, quinic acid, sucrose, α-D-glucose, and α-keto-glutaric acid can be used as sole carbon sources. Alanine, glycine, glutamate, asparagine, and meso-diaminopimelic acid were identified in the whole cell hydrolysates. The whole cell sugar profiles contained galactose, xylose, rhamnose and ribose. Polar lipids comprise diphosphatidylglycerol, phosphatidylglycerol, an unidentified aminophospholipid, and an unidentified phospholipid. The predominant menaquinone is MK-7(H_4_). The cellular fatty acids profile contains iso-C_16:0_, C_18:1_ω9*c*, and iso-C_16:0_ 2-OH as the major (>10%) components, with moderate (3–9%) amounts of iso-C_16:1 _H, C_17:1_ ω8*c* and 10-methyl C_17:0_. The G + C content of the genomic DNA is 71.9%. The type strain CPCC 204708^T^ (= DSM 105495 ^T^ = NBRC 112942^T^) was isolated from sandy soil collected from the Badain Jaran Desert in the Inner Mongolia autonomous region.

*Candidatus* “Solirubrobacter pratensis” (pra.ten’sis. L. fem. adj. *pratensis* growing in a meadow, referring to the isolation of the strain from grassland).

URHD0082 is temporarily proposed as the type genome for the species. The strain URHD0082 was isolated from the Mediterranean grassland soil. The accession number of the genome of the isolate URHD0082 is available in the DDBJ/ENA/Genbank database under accession AUEK00000000 and the Genbank accession number for the 16S rRNA gene sequence (extracted from the genome) is OQ674416. The genome of URHD0082 has the following characteristics: a draft genome of 6,640,086 bp, assembled from 28 qualified scaffolds, with total 6,557 genes, including 6,470 protein-coding genes and 87 RNA genes (consisting of 3 rRNA genes, 76 tRNA genes and 8 other RNA genes). The G + C content in the genomic DNA of URHD0082 is 72.2%.

## Data availability statement

The datasets presented in this study can be found in online repositories. The names of the repository/repositories and accession number(s) can be found in the article/Supplementary material.

## Ethics statement

The manuscript presents research on animals that do not require ethical approval for their study.

## Author contributions

Z-MJ: Conceptualization, Data curation, Formal analysis, Investigation, Validation, Visualization, Writing—original draft, Writing—review and editing. TM: Data curation, Investigation, Validation, Writing—original draft. YS: Formal analysis, Investigation, Validation, Writing—review and editing. JS: Resources, Validation, Investigation, Writing—review and editing. L-YY: Resources, Validation, Writing—review and editing. Y-QZ: Conceptualization, Data curation, Funding acquisition, Investigation, Methodology, Project administration, Supervision, Validation, Writing—original draft, Writing—review and editing.

## Footnotes

1
https://lpsn.dsmz.de/genus/solirubrobacter

2
https://cloud.majorbio.com/page/project/overview.html

3
https://www.ncbi.nlm.nih.gov/nuccore/AUEK00000000.1

4
https://www.ezbiocloud.net/

5
http://ggdc.dsmz.de/ggdc.php

6
http://www.cbcb.umd.edu/software/glimmer/

7
https://www.kegg.jp/

8
https://www.uniprot.org/

9
https://www.ebi.ac.uk/interpro/

10
https://antismash.secondarymetabolites.org/

11
https://rast.nmpdr.org/

12
https://tcdb.org/
