# Supplementary material for: Environmental distribution and genomic characteristics of Solirubrobacter, with proposal of two novel species
Source: Front Microbiol. 2023 Dec 1;14:1267771. doi: 10.3389/fmicb.2023.1267771 (PMC10722151; doi:10.3389/fmicb.2023.1267771)
Supplement: Supplementary file 1 [file Data_Sheet_1.pdf]

# Environmental Distribution and Genomic Characteristics of *Solirubrobacter*, with Proposal of Two Novel Species

Zhu-Ming Jiang[1, 2‡], Tong Mou[1, 2‡], Ye Sun[1],
Jing Su[1], Li-Yan Yu[1], Yu-Qin Zhang[1, 2*]

[1]Institute of Medicinal Biotechnology, Chinese Academy of Medical Sciences & Peking Union Medical College, Beijing 100050, P. R. China.

[2]State Key Laboratory of Dao-di Herb, Beijng, 100700, P. R. China

[*]Author for correspondence: Yu-Qin Zhang

Tel: +86-10-83167110

Fax: +86-10-83167110

E-Mail: yzhang@imb.pumc.edu.cn

[‡]Tong Mou shares the first authorship with Zhu-Ming Jiang.

**FIGURE S1 | Indole acetic acid (IAA) standard curve and absorbance values for fermentation broths of strains CPCC 204708$^T$, *S. phytolaccae* KCTC 29190$^T$, *S. taibaiensis* KCTC 29222$^T$, *S. pauli* JCM 13025$^T$, *S. ginsenosidimutans* DSM 21036$^T$, and *S. soli* DSM 22325$^T$ at 530 nm.** Orange, grey, yellow, purple, green, and red circles correspond to strains CPCC 204708$^T$, KCTC 29190$^T$, KCTC 29222$^T$, JCM 13025$^T$, DSM 21036$^T$, and DSM 22325$^T$, respectively.

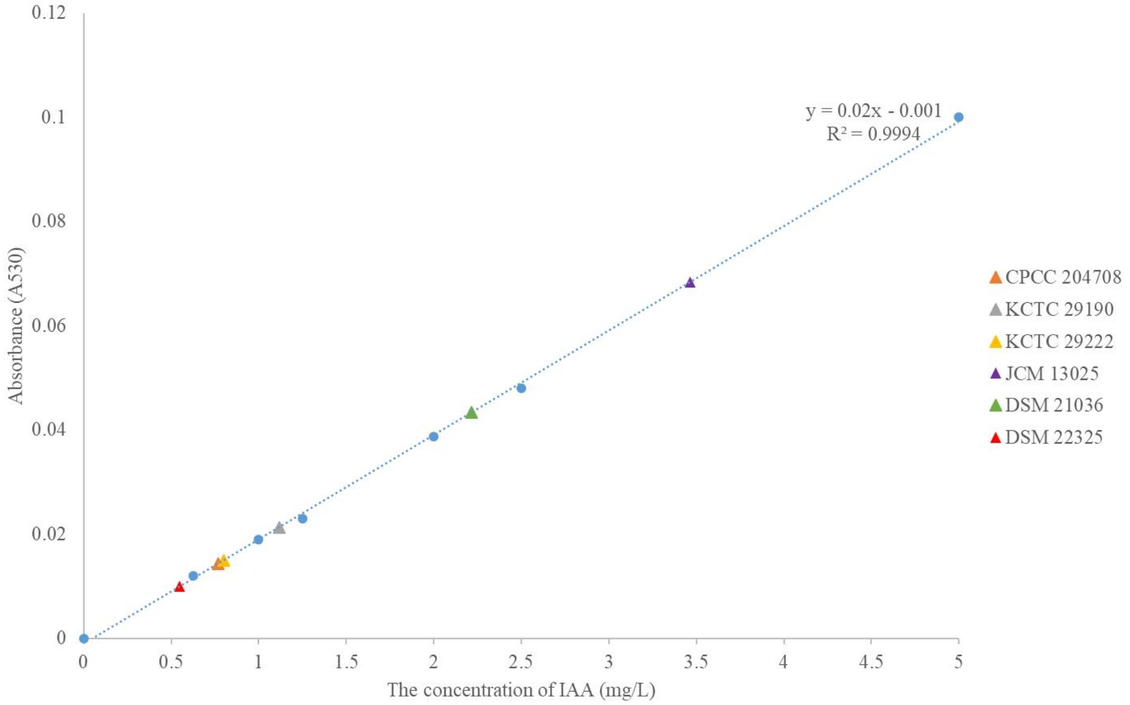

**FIGURE S2 | Neighbor-Joining phylogenetic tree of 16S rRNA gene sequences showing the relationships of strains CPCC 204708$^T$ and URHD0082 to other**

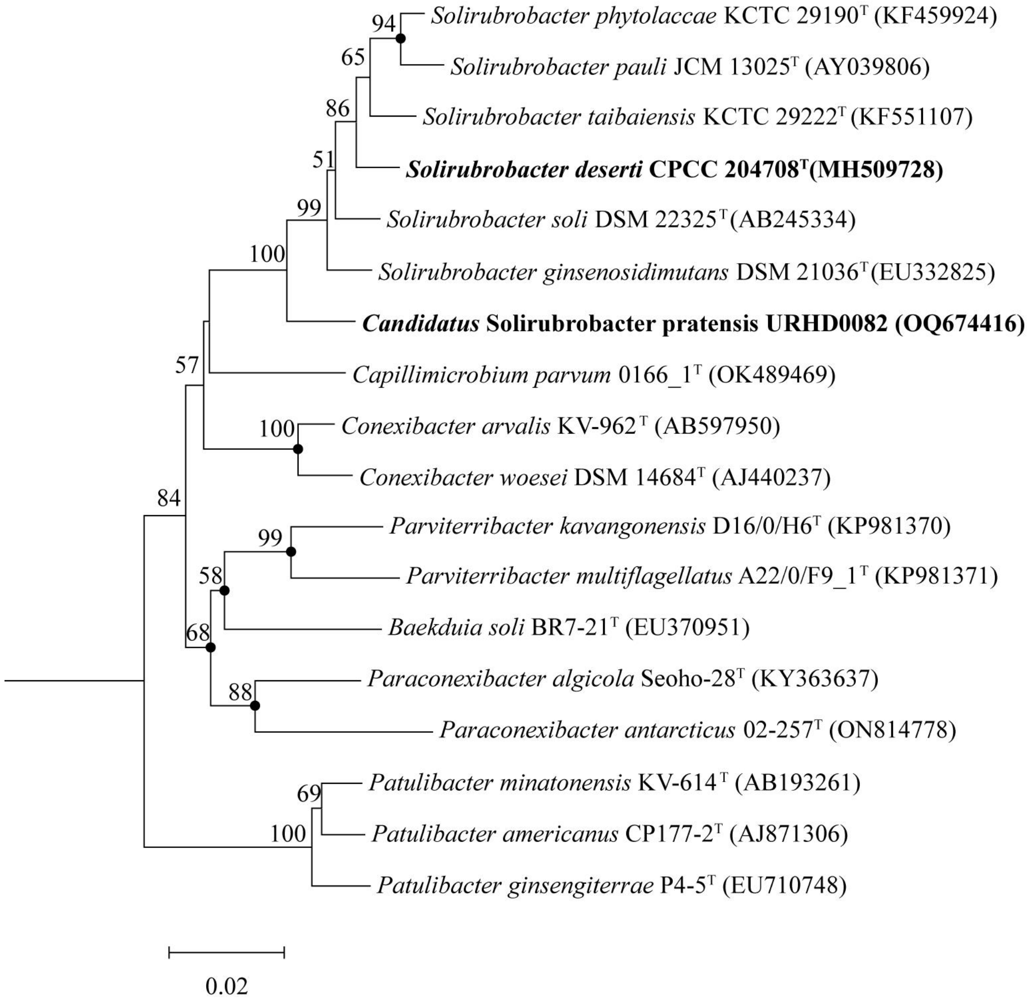


***Solirubrobacter* species.** *Micrococcus luteus* DSM 20030$^T$ was used as the outgroup (not shown). Filled circles indicate that the nodes were recovered in Maximum-Likelihood and Maximum Parsimony phylogenetic trees. Bootstrap values (above 50%) are shown as percentages of 1,000 replicates. Bar, 0.02 nt substitutions per site.

**FIGURE S3 | Phylogenetic tree from BPGA showing the relationships of newly proposed *Solirubrobacter* species with other *Solirubrobacter* species.** The phylogenetic tree is based on a binary gene presence/absence matrix (pan-matrix).

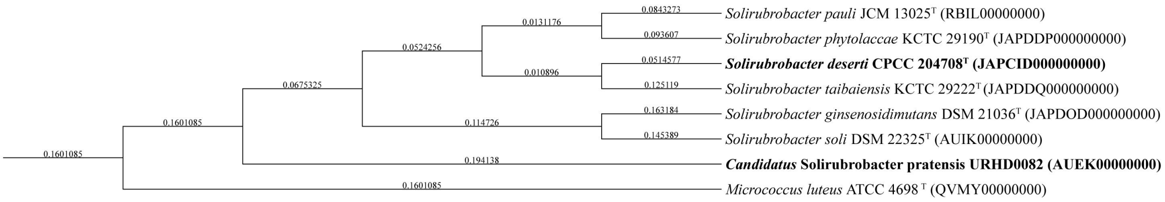

**FIGURE S4 | Transmission electron micrograph of strain CPCC 204708$^{T}$ grown on R2A medium at 28°C for 7 days.**

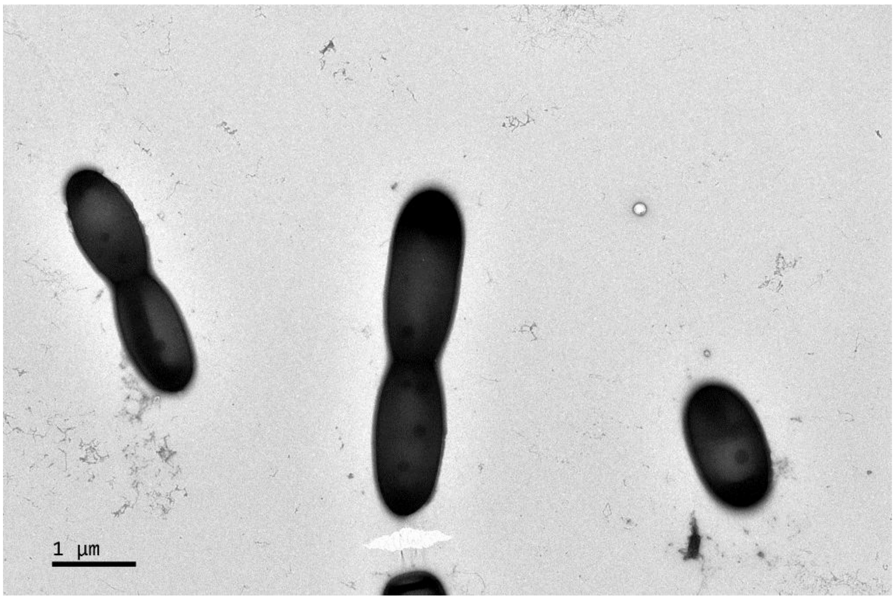

**FIGURE S5 | The indole-3-acetic acid (IAA) production pathway within the broader tryptophan metabolism pathways of strains CPCC 204708$^{T}$, KCTC 29190$^{T}$, KCTC 29222$^{T}$, JCM 13025$^{T}$, DSM 21036$^{T}$, DSM 22325$^{T}$, and URHD0082.** Squares in orange, green, red, blue, light blue, pink, and yellow correspond to strains CPCC 204708$^{T}$, KCTC 29190$^{T}$, KCTC 29222$^{T}$, JCM 13025$^{T}$, DSM 21036$^{T}$, DSM 22325$^{T}$, and URHD0082, respectively.

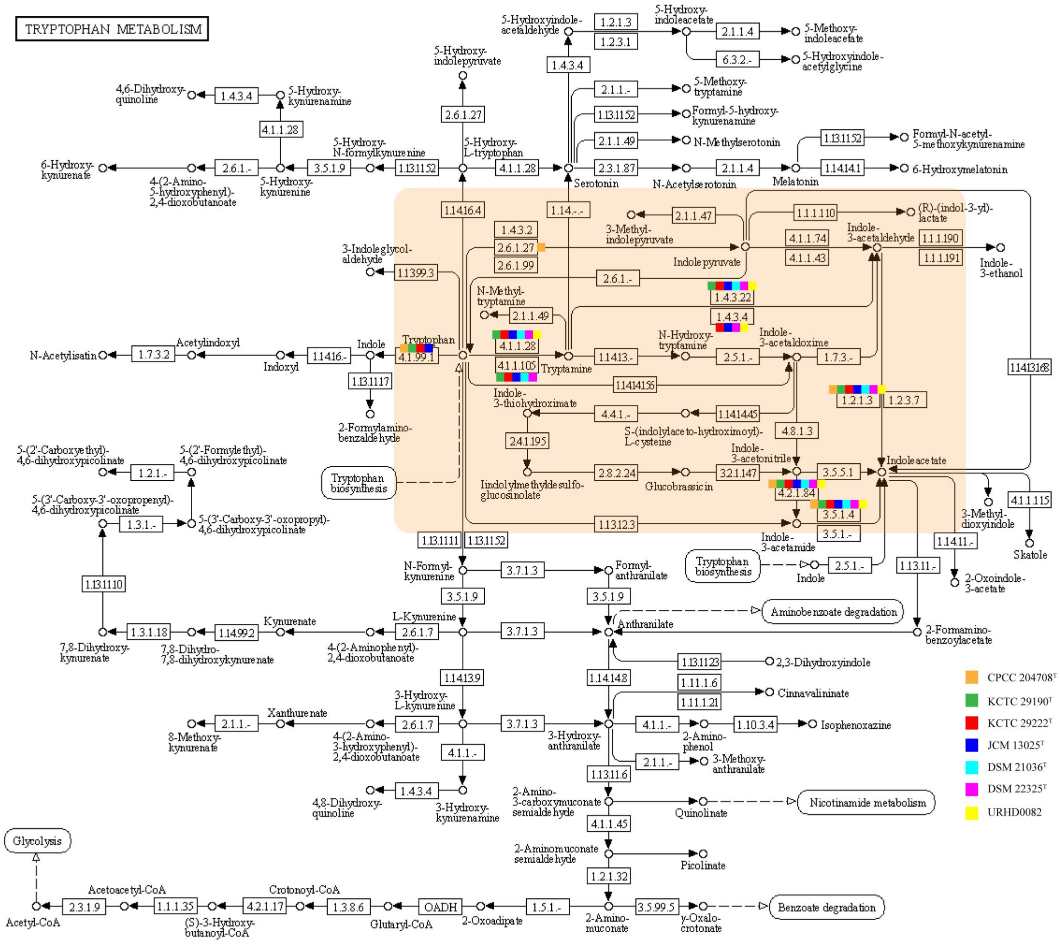

**FIGURE S6 | Polar lipid profiles of strain CPCC 204708$^T$ based on separation with two-dimensional TLC.**

This image was generated by spraying with phosphate stain reagent. DPG, diphosphatidylglycerol; PG, phosphatidylglycerol; PI, phosphatidylinositol; PIM, phosphatidylinositol mannosides; PL, unidentified phospholipid; APL, unidentified aminophospholipid.

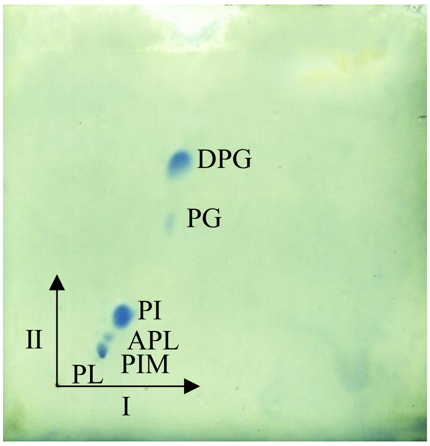

**FIGURE S7 | ABC transporters metabolism annotations of strain CPCC 204708$^{T}$ within the KEGG database.**

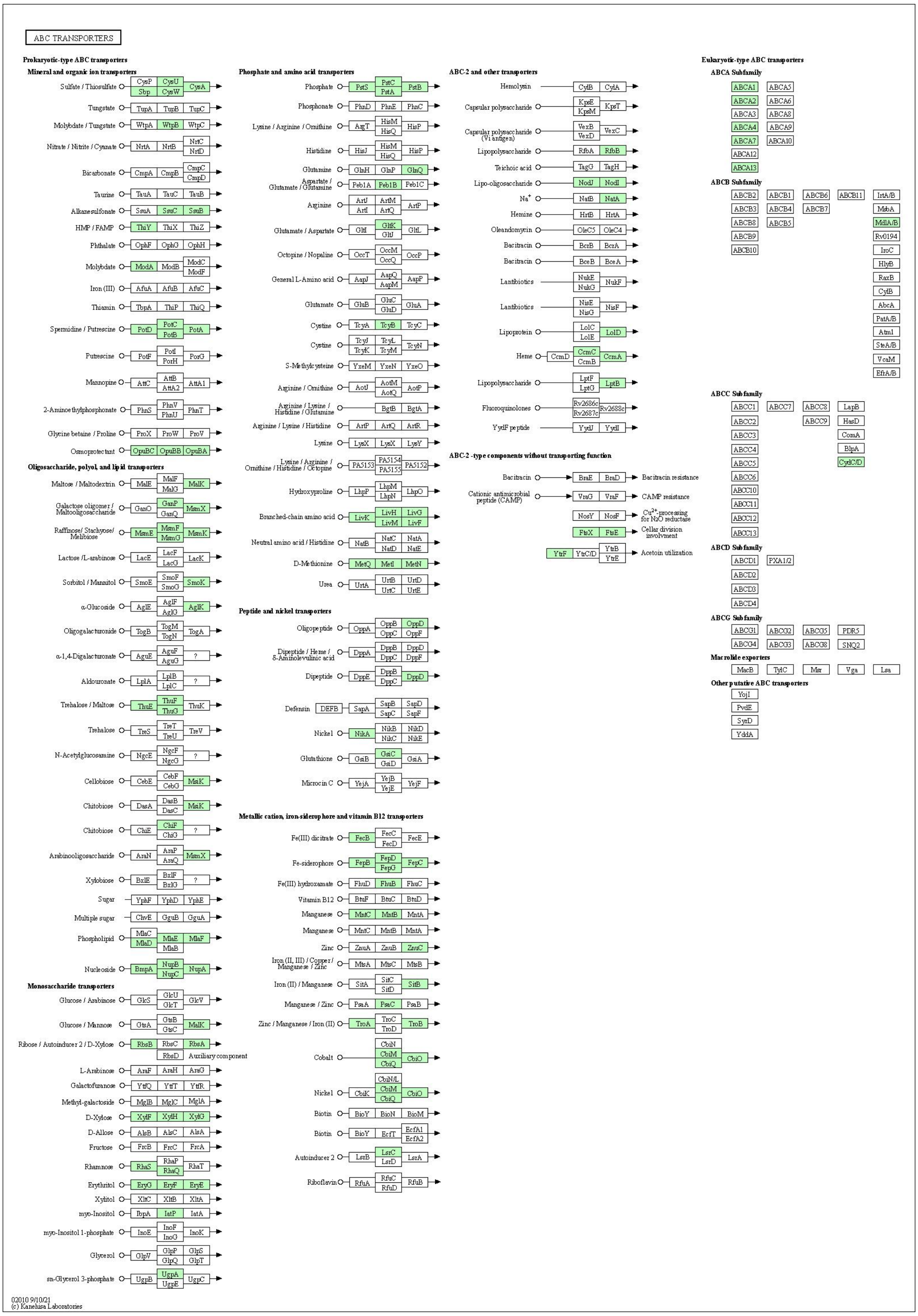

**FIGURE S8 | Pan-genomic analysis of the genomes from seven strains of *Solirubrobacter*.** (A) Abundances of gene families and corresponding homologous gene family conservation values. (B) Pan-genome gene family identification for *Solirubrobacter* based on increasing numbers of evaluated genomes.

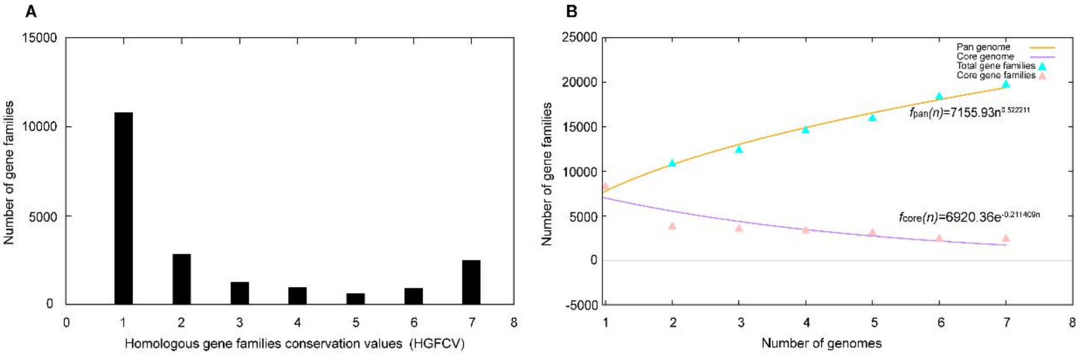

**FIGURE S9 | The nucleotide excision repair pathway of strain CPCC 204708$^T$ based on comparison to the KEGG database.**

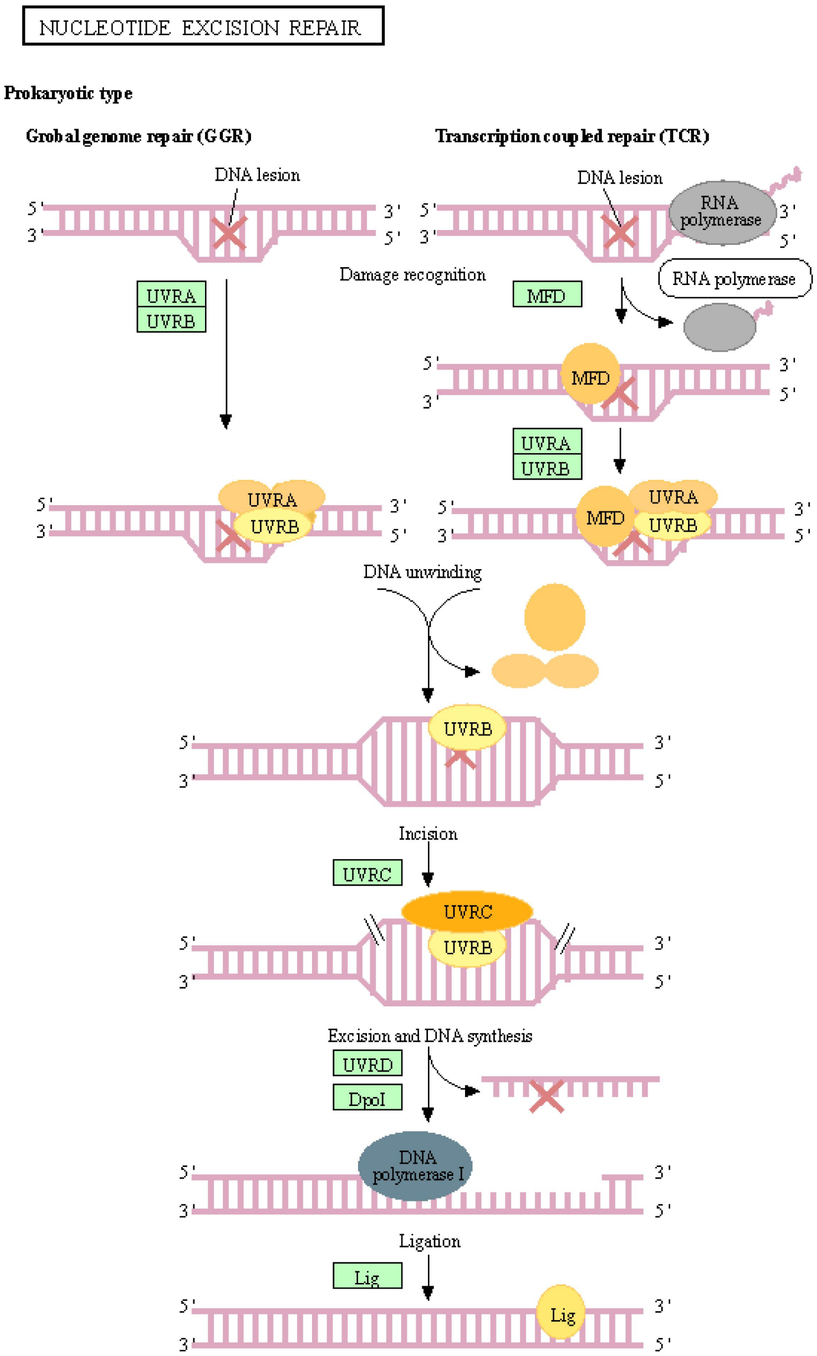

**FIGURE S10 | The homologous recombination pathway of strain CPCC 204708$^{T}$ based on comparison to the KEGG database.**

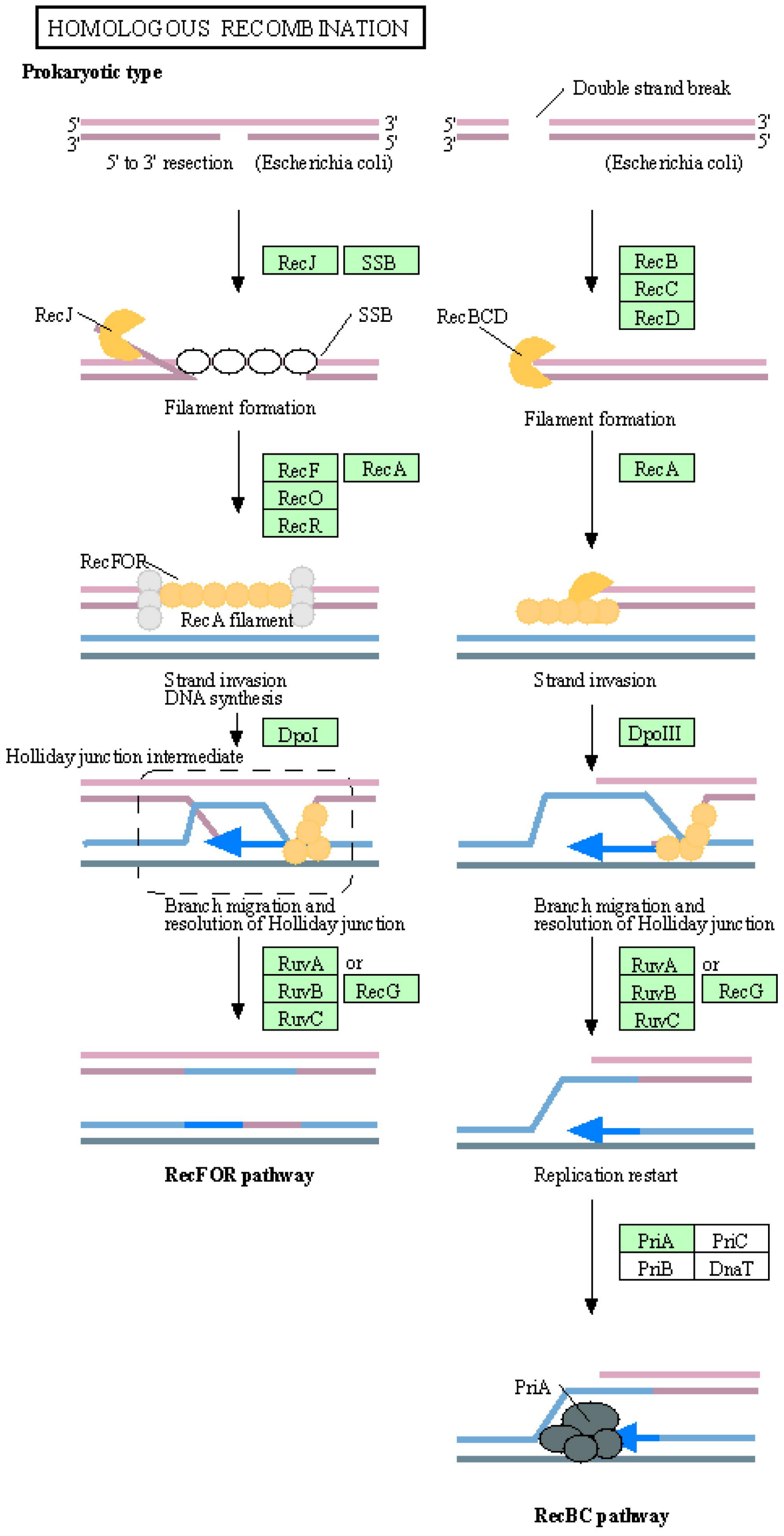

**Table S1 Information of samples collected in this study**

| Sample ID | Sample type | Latitude and Longitude | Altitude |
|---|---|---|---|
| IMB15101S–IMB15112S | Rhizosphere soil samples of herbs from high-altitude barren hills | 42°34′ N, 81°13′ E | 1,870–1,890 m |
| IMB21101S–IMB21112S | Rhizosphere soil samples of Yunnan Ethno-Medicinal plants | 24°35′ N, 101°13′ E | 1,980–2,200 m |
| IMB19101D–IMB19112D | Gurban Tungut Desert soil | 46°15' N, 87°33' E | 480–600 m |
| IMB15201D–IMB15212D | Tengger Desert | 37°28' N, 105°00' E | 1,330–1,700 m |
| IMB16101D–IMB16112D | Badain Jaran Desert | 39°56' N, 102°05' E | 1,140–1,790 m |
| IMB20301F–IMB20312F | Cow feces | 39°39′ N, 115°59′ E | 650–860 m |
| IMB20101F–IMB20112F | Crow feces samples | 39°46' N, 116°28' E | 35–40 m |
| IMB20201S–IMB20212S | Soil samples surrounding plateau Lakes | 26°03′ N, 105°34′ E | 1,990–2,100 m |
| IMB22101S–IMB22112S | Sediment soil samples | 30°16' N, 105°10' E | 220–260 m |
| IMB19301W–IMB19312W | Erhai Lake | 25°44' N, 100°12' E | 1,960–1,970 m |
| IMB14101E–IMB14112E | Phycosphere of agar cultures | 39°52' N, 116°23' E | 35 m |

**Table S2 Pairwise 16S rRNA gene similarity, average nucleotide identity (ANI), and digital DNA-DNA hybridization (dDDH) values among strain CPCC 204708$^{T}$ and other *Solirubrobacter*.**

| | CPCC 204708$^{T}$ | *S. phytolaccae* KCTC 29190$^{T}$ | *S. taibaiensis* KCTC 29222$^{T}$ | *S. ginsenosidimutans* DSM 21036$^{T}$ | *S. pauli* JCM 13025$^{T}$ | *S. soli* DSM 22325$^{T}$ | *Candidatus* S. pratensis sp. nov. URHD0082 |
|---|---|---|---|---|---|---|---|
| CPCC 204708$^{T}$ | (100.0/100.0/100.0) | / | / | / | / | / | / |
| *S. phytolaccae* KCTC 29190$^{T}$ | (98.2/83.4/26.4) | (100.0/100.0/100.0) | / | / | / | / | / |
| *S. taibaiensis* KCTC 29222$^{T}$ | (98.1/84.4/27.6) | (98.8/83.5/26.4) | (100.0/100.0/100.0) | / | / | / | / |
| *S. ginsenosidimutans* DSM 21036$^{T}$ | (97.4/78.2/21.1) | (97.1/78.3/21.2) | (97.9/77.9/20.9) | (100.0/100.0/100.0) | / | / | / |
| *S. pauli* JCM 13025$^{T}$ | (97.6/83.5/26.5) | (98.4/86.9/31.9) | (97.7/83.1/26.0) | (96.1/78.3/21.2) | (100.0/100.0/100.0) | / | / |
| *S. soli* DSM 22325$^{T}$ | (97.7/78.6/21.6) | (97.0/78.4/21.3) | (98.0/78.1/21.1) | (98.6/82.9/25.8) | (97.0/78.5/21.4) | (100.0/100.0/100.0) | / |
| *Candidatus* S. pratensis sp. nov. URHD0082 | (97.3/77.3/21.0) | (96.8/77.4/20.9) | (96.9/76.9/20.5) | (97.5/78.1/21.4) | (95.9/77.5/21.1) | (97.4/78.1/21.3) | (100.0/100.0/100.0) |

Note: Values in parentheses are 16S rRNA gene similarity, ANI, and dDDH values, from left to right.

**Table S3 Fatty acid profiles of strain CPCC 204708$^T$ and the type strains of other *Solirubrobacter* species.**

| Fatty acid | 1 | 2 | 3 | 4 | 5 | 6 |
|---|---|---|---|---|---|---|
| ***anteiso*-branched fatty acid** | | | | | | |
| anteiso-$C_{15:0}$ | 2.7 | - | - | - | - | - |
| anteiso-$C_{17:0}$ | 0.6 | TR | TR | TR | TR | 2.6 |
| ***iso*-branched fatty acid** | | | | | | |
| iso-$C_{15:0}$ | 1.5 | - | - | - | - | - |
| iso-$C_{16:0}$ | 31.6 | 31.9 | 37.4 | 35.8 | 26.8 | 18.4 |
| iso-$C_{16:1}$ H | 8.2 | 1.4 | 2 | 1.4 | TR | 1.5 |
| iso-$C_{16:1}$ F | 1.4 | - | - | - | - | - |
| iso-$C_{17:0}$ | TR | - | - | - | - | - |
| iso-$C_{18:0}$ | - | 1.1 | - | - | 2.5 | 2 |
| **Straight-chain fatty acid** | | | | | | |
| $C_{14:0}$ | - | 1.4 | TR | 1.4 | 2.3 | 2.6 |
| $C_{15:0}$ | - | 1.8 | 1.7 | 1.5 | 3.0 | 2.4 |
| $C_{16:0}$ | - | 4.8 | 6.1 | 4.5 | 3.8 | 3.9 |
| $C_{17:0}$ | - | 3.2 | 2.4 | 2.0 | 6.1 | 3.9 |
| $C_{18:0}$ | 1.4 | 1.7 | 1.9 | 1.0 | 2.2 | 2.5 |
| $C_{19:0}$ | 0.8 | TR | - | TR | 1.4 | 1.7 |
| 10-Methyl $C_{17:0}$ | 5.2 | 1.3 | 2.3 | 1.9 | 1.4 | - |
| 10-Methyl $C_{18:0}$ | - | 2.9 | - | 2.6 | 7.9 | - |
| 10-Methyl $C_{19:0}$ | - | 2.2 | - | - | 5.2 | - |
| **Hydroxy fatty acids** | | | | | | |
| iso-$C_{14:0}$ 3-OH | - | 1.9 | - | - | - | - |
| iso-$C_{16:0}$ 2-OH | 13.7 | - | - | - | - | - |
| **0thers** | | | | | | |
| $C_{16:1}$ $\varpi 7c$ | 1.2 | - | - | - | - | - |
| $C_{17:1}$ $\varpi 11c$ | - | 6.0 | 8.6 | 11.0 | 2.6 | 4.7 |
| $C_{17:1}$ $\varpi 9c$ | - | 11.2 | 15.3 | 7.8 | 6.6 | 4.4 |
| $C_{17:1}$ $\varpi 8c$ | 6.0 | - | - | - | - | - |
| $C_{18:3}$ $\varpi 12,6,4c$ | 1.2 | - | - | - | - | - |
| $C_{18:3}$ $\varpi 12,9,6c$ | - | 8.1 | 3.1 | 7.2 | 9.5 | 13.7 |
| $C_{18:1}$ $\varpi 9c$ | 21.7 | 14.2 | 13.3 | 17.7 | 13.4 | 14.2 |
| $C_{20:1}$ $\varpi 11c$ | - | - | - | - | TR | 1.3 |

Note: 1, CPCC 204708$^T$ (data from this study); 2, *S. phytolaccae* KCTC 29190$^T$ (Wei et al., 2014); 3, *S. pauli* JCM 13025$^T$ (Singleton et al., 2003); 4, *S. taibaiensis* KCTC 29222$^T$ (Zhang et al., 2014); 5, *S. ginsenosidimutans* DSM 21036$^T$ (An et al., 2011); 6, *S. soli* DSM 22325$^T$ (Kim et al., 2007). TR: trace amount (< 0.5%); -, not detected.

**Table S4 Secondary metabolite biosynthesis gene clusters predicted in the genomes of seven *Solirubrobacter* strains.**

| Genomic location | Type | Secondary metabolite synthesis gene cluster | Similarity |
|---|---|---|---|
| **CPCC 204708$^T$** | | | |
| Scaffold4_1Region 5.1 | NAPS-independent-siderophore | / | / |
| Scaffold4_1Region 5.2 | RiPP-like | / | / |
| Scaffold11_1Region 12.1 | oligosaccharide | Accramycin A Polyketide | 5% |
| Scaffold14_1Region 15.1 | RRE-containing | microansamycin | / |
| Scaffold25_1Region 27.1 | RiPP-like | / | / |
| Scaffold25_1Region 27.2 | Redox-cofactor | NRP: Lipopeptide: $Ca^+$-dependent lipopeptide | 7% |
| Scaffold26_1Region 28.1 | Lassopeptide | / | 12% |
| Scaffold31_1Region 33.1 | LAP/ thiopeptide | / | / |
| Scaffold47_1Region 51.1 | terpene | / | / |
| Scaffold54_1Region 58.1 | terpene | NRP: Lipopeptide: $Ca^+$-dependent lipopeptide | 7% |
| ***S. phytolaccae* KCTC 29190$^T$** | | | |
| Scaffold1_1Region 1.1 | Other | / | / |
| Scaffold4_1Region 4.1 | RiPP-like | / | / |
| Scaffold8_1Region 8.1 | Lassopeptide | / | / |
| Scaffold9_1Region 9.1 | NAPAA | / | / |
| Scaffold22_1Region 22.1 | Terpene | / | / |
| Scaffold25_1Region 25.1 | Phenazine | Lomofungin | 39% |
| Scaffold26_1Region 26.1 | Terpene | NRP: Lipopeptide: $Ca^+$-dependent lipopeptide | 7% |
| Scaffold65_1Region 65.1 | LAP/ thiopeptide | / | / |
| Scaffold113_1Region 113.1 | NAPS-independent-siderophore | / | / |
| Scaffold129_1Region 129.1 | Redox-cofactor | / | / |
| ***S. pauli* JCM 13025$^T$** | | | |
| NZ_RBIL01000001.1 Region 1.1 | RiPP-like | / | / |
| NZ_RBIL01000001.1 Region 1.2 | Lanthipeptide-class-iv | Tiancimycin | 5% |
| NZ_RBIL01000001.1 Region 1.3 | Terpene | / | / |
| NZ_RBIL01000001.1 Region 1.4 | NAPAA | / | / |
| NZ_RBIL01000002.1 Region 2.1 | Terpene | NRP: Lipopeptide: $Ca^+$-dependent lipopeptide | 7% |
| NZ_RBIL01000002.1 | Other | / | / |

| | | | |
|---|---|---|---|
| Region 2.2 | | | |
| NZ_RBIL01000002.1 Region 2.3 | NAPS-independent-siderophore | Schizokinen | 20% |
| NZ_RBIL01000002.1 Region 2.4 | Redox-cofactor | / | / |
| NZ_RBIL01000003.1 Region 3.1 | Other | Actagardine RiPP: Lanthipeptide | 9% |
| NZ_RBIL01000003.1 Region 3.2 | RRE-containing | / | / |
| ***Candidatus* S. pratensis sp. nov. URHD0082** | | | |
| NZ_KE384480.1 Region 1.1 | Redox-cofactor | / | 13% |
| NZ_KE384480.1 Region 1.2 | Terpene | / | / |
| NZ_KE384480.1 Region 1.3 | Ranthipeptide | / | / |
| NZ_KE384481.1 Region 3.1 | RiPP-like | / | / |
| NZ_AUEK01000014.1 Region 12.1 | Terpene | NRP: Lipopeptide: Ca$^{+}$-dependent lipopeptide | 10% |
| ***S. ginsenosidimutans* DSM 21036$^{T}$** | | | |
| Scaffold4_1Region 4.1 | Redox-cofactor | Lankacidin C/ NRP+Polyketide | 13% |
| Scaffold5_1Region 5.1 | Indole | / | / |
| Scaffold7_1Region 7.1 | Indole | / | / |
| Scaffold8_1Region 8.1 | RiPP-like | / | / |
| Scaffold8_1Region 8.2 | Redox-cofactor | / | / |
| Scaffold9_1Region 9.1 | NAPS-independent-siderophore | Schizokinen | 20% |
| Scaffold10_1Region 10.1 | RiPP-like | / | / |
| Scaffold12_1Region 13.1 | Other | / | / |
| Scaffold14_1Region 15.1 | Other | / | / |
| Scaffold24_1Region 25.1 | Terpene | NRP: Lipopeptide: Ca$^{+}$-dependent lipopeptide | 7% |
| Scaffold27_1Region 28.1 | RRE-containing | / | / |
| Scaffold49_1Region 50.1 | terpene | / | / |
| Scaffold69_1Region 70.1 | RRE-containing | / | / |
| Scaffold134_1Region 135.1 | RiPP-like | / | / |
| ***S. taibaiensis* KCTC 29222$^{T}$** | | | |
| Scaffold1_1Region 1.1 | T1PKS/ hglE-KS | Linfuranone B/ linfuranone C/ Polyketide | 13% |
| Scaffold4_1Region 4.1 | Lassopeptide | / | / |
| Scaffold8_1Region 8.1 | Terpene | / | / |
| Scaffold12_1Region 12.1 | RiPP-like | / | / |
| Scaffold27_1Region 27.1 | Other | / | / |
| Scaffold51_1Region 51.1 | Terpene | Kitasetaline/ Alkaloid | 40% |
| Scaffold67_1Region 67.1 | Redox-cofactor | / | / |
| Scaffold79_1Region 79.1 | NAPS-independent-siderophor | / | / |

| | | | |
|---|---|---|---|
| | e | | |
| ***S. soli* DSM 22325$^{T}$** | | | |
| NZ_KE384069.1 Region 2.1 | Terpene | NRP: Lipopeptide: Ca$^{+}$-dependent lipopeptide | 10% |
| NZ_AUIK01000008.1 Region 5.1 | Lanthipeptide-class-iv | / | / |
| NZ_AUIK01000010.1 Region 7.1 | NAPS-independent-siderophore | / | / |
| NZ_AUIK01000010.1 Region 7.2 | Redox-cofactor | / | / |
| NZ_AUIK01000010.1 Region 7.3 | RiPP-like | / | / |
| NZ_KE384072.1 Region 8.1 | Other | Fulvuthiacene A/ Fulvuthiacene B NRP+Polyketide | 8% |
| NZ_AUIK01000020.1 Region 12.1 | Other | / | / |
| NZ_AUIK01000020.1 Region 12.2 | NRPS | / | / |
| NZ_AUIK01000023.1 Region 15.1 | Other | / | / |
| NZ_AUIK01000029.1 Region 21.1 | Lassopeptide | / | / |
| NZ_AUIK01000035.1 Region 27.1 | Indole | Macrotermycins | 11% |

Note: /: not detected.
